# Myoglobin‐mediated lipid shuttling increases adrenergic activation of brown and white adipocyte metabolism and is as a marker of thermogenic adipocytes in humans

**DOI:** 10.1002/ctm2.1108

**Published:** 2022-12-08

**Authors:** Lisa Christen, Helen Broghammer, Inka Rapöhn, Kevin Möhlis, Christian Strehlau, Aleix Ribas‐Latre, Claudia Gebhardt, Lisa Roth, Kerstin Krause, Kathrin Landgraf, Antje Körner, Kerstin Rohde‐Zimmermann, Anne Hoffmann, Nora Klöting, Adhideb Ghosh, Wenfei Sun, Hua Dong, Christian Wolfrum, Tienush Rassaf, Ulrike B. Hendgen‐Cotta, Michael Stumvoll, Matthias Blüher, John T. Heiker, Juliane Weiner

**Affiliations:** ^1^ Helmholtz Institute for Metabolic, Obesity and Vascular Research (HI‐MAG) of the Helmholtz Zentrum München at the University of Leipzig and University Hospital Leipzig Leipzig Germany; ^2^ Medical Department III ‐ Endocrinology Nephrology Rheumatology University of Leipzig Medical Center Leipzig Germany; ^3^ Center for Pediatric Research Leipzig (CPL) University Hospital for Children and Adolescents Medical Faculty University of Leipzig Leipzig Germany; ^4^ Institute of Food Nutrition and Health ETH Zurich Schwerzenbach Switzerland; ^5^ Department of Cardiology and Vascular Medicine West German Heart and Vascular Center Medical Faculty University of Duisburg‐Essen Essen Germany; ^6^ Institute of Biochemistry, Faculty of Life Sciences University of Leipzig Leipzig Germany

**Keywords:** energy expenditure, hemoprotein, metabolism, obesity, oxphos, uncoupling protein 1

## Abstract

**Background:**

Recruitment and activation of brown adipose tissue (BAT) results in increased energy expenditure (EE) via thermogenesis and represents an intriguing therapeutic approach to combat obesity and treat associated diseases. Thermogenesis requires an increased and efficient supply of energy substrates and oxygen to the BAT. The hemoprotein myoglobin (MB) is primarily expressed in heart and skeletal muscle fibres, where it facilitates oxygen storage and flux to the mitochondria during exercise. In the last years, further contributions of MB have been assigned to the scavenging of reactive oxygen species (ROS), the regulation of cellular nitric oxide (NO) levels and also lipid binding. There is a substantial expression of MB in BAT, which is induced during brown adipocyte differentiation and BAT activation. This suggests MB as a previously unrecognized player in BAT contributing to thermogenesis.

**Methods and Results:**

This study analyzed the consequences of MB expression in BAT on mitochondrial function and thermogenesis in vitro and in vivo. Using MB overexpressing, knockdown or knockout adipocytes, we show that expression levels of MB control brown adipocyte mitochondrial respiratory capacity and acute response to adrenergic stimulation, signalling and lipolysis. Overexpression in white adipocytes also increases their metabolic activity. Mutation of lipid interacting residues in MB abolished these beneficial effects of MB. In vivo, whole‐body MB knockout resulted in impaired thermoregulation and cold‐ as well as drug‐induced BAT activation in mice. In humans, *MB* is differentially expressed in subcutaneous (SC) and visceral (VIS) adipose tissue (AT) depots, differentially regulated by the state of obesity and higher expressed in AT samples that exhibit higher thermogenic potential.

**Conclusions:**

These data demonstrate for the first time a functional relevance of MBs lipid binding properties and establish MB as an important regulatory element of thermogenic capacity in brown and likely beige adipocytes.

## Graphical abstract

- Myoglobin (MB) is highly expressed in brown adipose tissue (BAT), its expression level increases with adipocyte differentiation and it is temperature dependently regulated in vivo.
- Expression levels of MB in brown and white adipocytes control mitochondrial respiratory capacity and increase acute response to adrenergic signalling and lipolysis.
- Effects of MB in adipocytes seem mainly mediated by its interaction with lipids to increase substrate flux to mitochondria.
- In mice, whole‐body knockout of MB results in impaired thermoregulation and BAT activation.
- In human white adipose tissue depots, MB is differentially expressed in obesity and correlates with markers of AT browning.

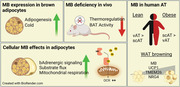

## 1 INTRODUCTION

The main function of brown adipose tissue (BAT) is to maintain body temperature. This is achieved by uncoupling oxidative phosphorylation from ATP synthesis via expression of its hallmark protein uncoupling protein 1 (UCP1) resulting in heat production, a process termed thermogenesis. Therefore, BAT is a highly metabolically active organ with a considerable contribution to whole‐body energy expenditure (EE) in small mammals. The rediscovery of functional BAT in adult humans^1^, ^2^, ^3^ provided new perspectives for the treatment of obesity and associated diseases^4^ and sparked research on the role of BAT and uncoupling in human thermal biology and body weight regulation.

BAT thermogenesis is under the control of the sympathetic nervous system and activated by adrenergic stimulation after cold exposure or food intake.^5^ Furthermore, adipocytes with typical features of brown adipocytes emerge in white adipose tissue (WAT) after cold exposure and were termed “brite” or “beige” adipocytes. These cell types either derive from phenotypic trans‐differentiation or de novo differentiation of white or beige progenitor cells.^6^, ^7^

BAT exhibits high plasticity that is required for extensive recruitment and remodelling processes upon activation, for example, when the ambient temperature falls below the thermoneutral temperature. Increased thermogenic capacity after adrenergic activation is predominantly achieved by an increase in brown adipocyte number, resulting in higher UCP1 protein expression, and driven by enhanced proliferation (reviewed in^8^) and fueled by angiogenesis.^9^ BAT mainly utilizes lipids, but also substantial amounts of glucose to generate heat,^10^ with further substrates such as branched‐chain amino acids and tricarboxylic acid (TCA) cycle metabolites contributing to full thermogenic capacity.^11^ The thermogenic process requires a continuous flux of oxygen and lipids as well as carbohydrate substrates to BAT mitochondria.

Brown adipocytes are developmentally related to muscle cells and express muscle‐like gene and mitochondrial proteome signatures.^12^, ^13^ The hemoprotein myoglobin (MB) is a cytoplasmic protein highly expressed in cardiac and skeletal muscle where it contributes to cellular oxygen storage and diffusion to the mitochondria under conditions of high oxygen demand.^14^ MB further regulates mitochondrial activity and capacity via modulation of cellular nitric oxide (NO) levels^15^ or as a scavenger of reactive oxygen species (ROS).^16^ In myocardial cells, MB is localized in mitochondria,^17^, ^18^ where it may directly interact with respiratory chain complex IV.^19^ Recent studies also demonstrated fatty acid (FA) and acyl carnitine binding especially to oxygenated MB suggesting a potential role in lipid metabolism.^20^, ^21^, ^22^ Mb‐deficient mice (Mb‐KO) show ectopic fat deposition with increased lipid accumulation containing preferentially palmitic and oleic FA in the heart,^23^ which is accompanied by a switch from FA utilization to glucose oxidation in cardiac muscle.^24^

Intriguingly, MB is highly expressed in murine BAT and expression is further increased after cold exposure.^25^, ^26^ In BAT, MB can serve multiple purposes to support and facilitate mitochondrial respiration and thermogenic capacity. First, by increasing oxygen flux for the oxidation of substrates in the mitochondria. In addition, MB may contribute as a ROS scavenger to protect BAT against ROS accumulation and subsequent oxidative damage during high respiratory rates for thermogenesis. Two very recent studies initially proposed a functional role of MB in brown adipocyte metabolism. These studies reported initial phenotypic and metabolic alterations in MB‐deficient BAT of Mb‐KO mice fed a standard chow^27^ or a high fat diet.^28^ Mb‐KO mice were heavier, with larger BAT lipid droplets and lower expression levels of genes and proteins related to oxidative phosphorylation and thermogenesis. Functionally, BAT explants from Mb‐KO mice showed reduced mitochondrial respiratory capacity.^27^ These data suggested MB as an essential regulator of the full thermogenic capacity of BAT. Yet, the first experimental proof of functional consequences of MB expression on the adipocyte level and during cold‐induced BAT activation is lacking. Second, the important question of how MB may support BAT metabolism and thermogenesis mechanistically remains unclear.

In this study, using immortalized and primary brown and white adipocyte cell models, we demonstrate the direct effect of MB expression levels on mitochondrial respiration, lipolysis and adrenergic activation via genetic manipulations including *Mb* knockout, knockdown or overexpression. We confirm significant induction of MB gene and protein expression in BAT of cold‐exposed mice and during adipocyte differentiation. Using multiple adipocyte cell models with varying levels of MB expression, we show that MB controls brown adipocyte mitochondrial respiratory capacity and acute response to adrenergic stimulation, signalling and lipolysis. Mutation of lipid interacting residues in MB abolished these beneficial effects of MB. We further show the consequences of Mb‐deficiency on thermoregulation at different housing temperatures in vivo, using an established Mb‐KO mouse model.^29^ Finally, in human transcriptome data we demonstrate for the first time that WAT *MB* expression is differentially regulated in obesity and correlates with *UCP1* and other markers of adipose tissue (AT) browning, suggesting functional importance of MB expression in human AT.

## 2 RESULTS

### 2.1 BAT MB expression is increased in cold‐exposed mice and during brown adipocyte differentiation

**FIGURE 1:**
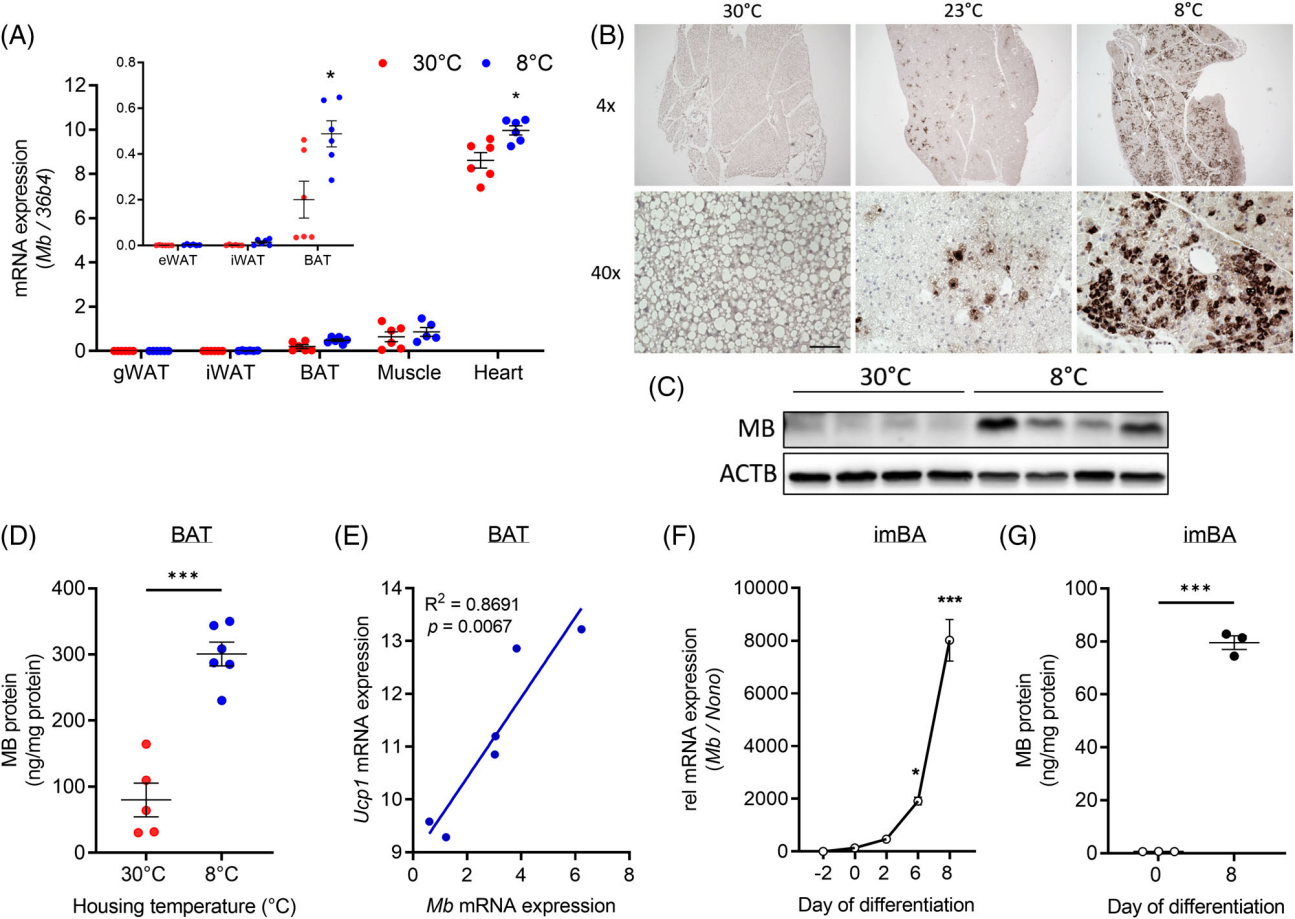
Temperature‐ and differentiation‐dependence of MB expression in BAT and during differentiation of brown adipocytes. (A) *Mb* mRNA expression in gonadal (gWAT), inguinal (iWAT) and brown adipose tissue (BAT), muscle (quadriceps femoris) and heart of female C57BL/6N mice housed either at 30°C or 8°C for 1 week (*n* = 6 per group). For better appreciation, *Mb* expression in AT depots is also presented as inset. (B) Representative images of MB immunohistochemistry in BAT from female mice as in (A). Scale bar: 100 μm. (C) Western blot analysis of MB expression in BAT of male C57BL/6N mice housed either at 30°C or 8°C (*n* = 4/4). MB expression was normalized to ACTB. (D) MB protein concentration measured by ELISA in BAT lysates from male NMRI WT mice housed either at 30°C or 8°C for 1 week (*n* = 6 per group). (E) Correlation of *Mb* and *Ucp1* mRNA expression in female C57BL/6N mice housed at 8°C for 1 week (*n* = 6) determined by simple linear regression. (F) *Mb* mRNA expression in imBA cells during differentiation relative to day 0 (pre‐induction) and normalized to *Nono*. (G) MB protein concentration in imBA before and after differentiation measured by ELISA. Data are presented as mean ± SEM of at least three independent experiments, if not stated otherwise. Statistical significance was evaluated by uncorrected multiple unpaired t‐tests (A), unpaired *t*‐test (D, G) or by one‐way ANOVA with Dunnett's (F) post hoc test. *p‐value < .05, ***p‐value < .001. Scale bar: 100 μm.

We first investigated temperature‐ and differentiation‐dependent changes in MB gene and protein expression in BAT and brown adipocytes in vivo and in vitro. In male and female C57BL/6N mice, we found a significant temperature‐dependent increase in MB mRNA and protein expression in BAT of cold‐exposed mice when compared to mice housed at thermoneutrality (Figure 1A–C and Figure S1A–C). Morphologically, cold‐induced BAT remodelling is characterized by the appearance of multilocular adipocytes with small lipid droplets. After cold exposure, distinct MB‐expressing adipocytes emerge in dense patterns and are evenly distributed within the tissue. *Mb* gene expression in the heart was also increased at 8°C (Figure 1A). In line, MB enzyme‐linked immunosorbent assay (ELISA) measurements from BAT lysates of NMRI wildtype (WT) mice housed at 30°C and 8°C, showed highly significant induction of MB protein content in cold‐exposed mice (Figure 1D). *Mb* expression significantly correlated with *Ucp1* expression (Figure 1E) in BAT. Furthermore, and confirming previous findings,^27^ we observed a strong increase of MB mRNA and protein expression during the differentiation of immortalized brown adipocytes (imBA), with the highest levels in differentiated brown adipocytes (Figure 1F,G).

### 2.2 Regulation of adipocyte *Mb* gene expression

**FIGURE 2:**
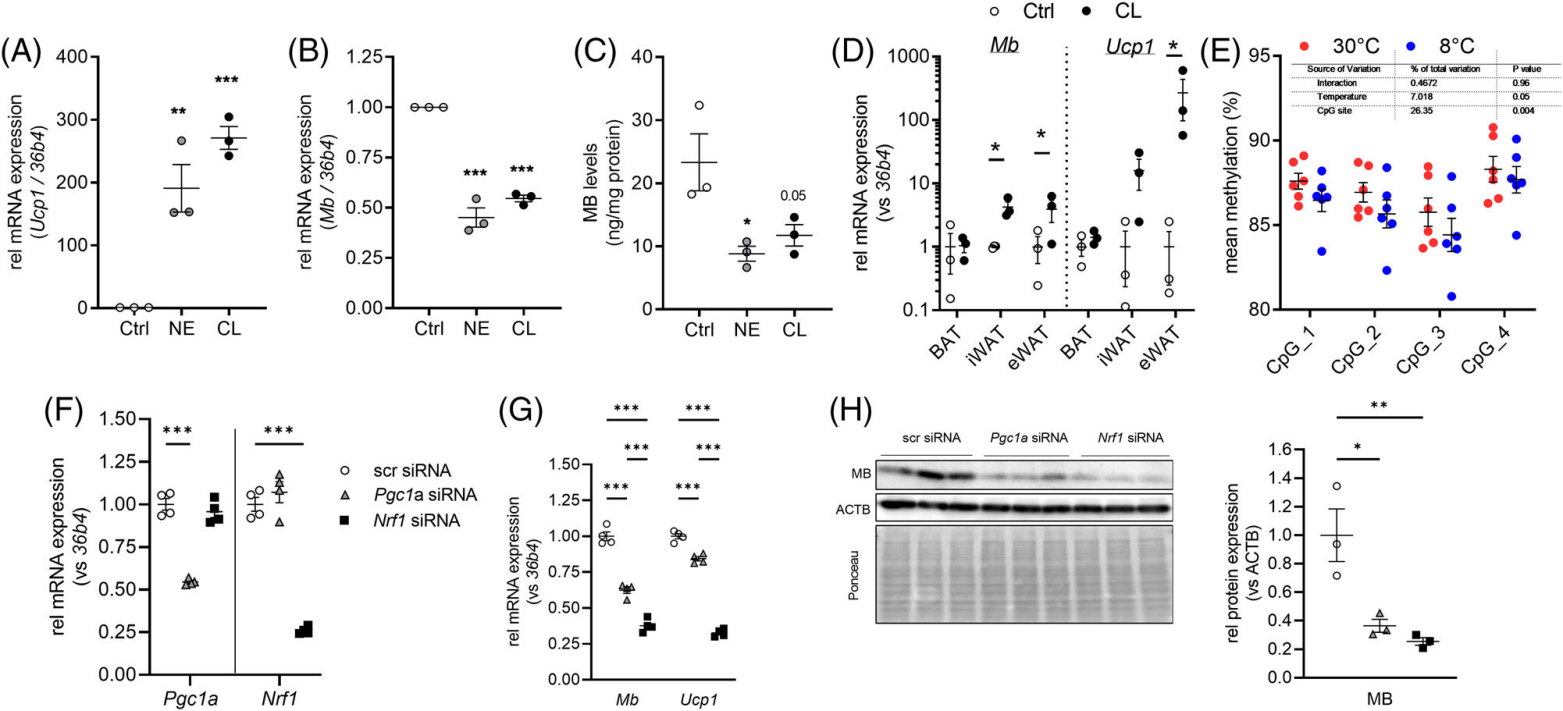
Regulatory mechanisms of MB expression in imBA and BAT. Effects of norepinephrine (NE) and CL316,243 (CL) on *Ucp1* (A) and *Mb* (B) mRNA and MB protein (C) expression in differentiated imBA cells. (D) *Mb* and *Ucp1* mRNA expression in BAT, as well as epididymal and inguinal WAT of control and CL316,243 (CL) treated male C57BL/6N mice (*n* = 3 per group). Gene expression is relative to controls and normalized to *36b4*. (E) Promoter methylation at CpG sites within a putative NRF1 binding site in BAT of mice housed either at 30°C or 8°C (*n* = 6 per group). (F) *Pgc1a* and *Nrf1* mRNA expression after respective siRNA‐mediated knockdown in differentiated imBA cells (*n* = 4/4/4). *Ucp1* and *Mb* mRNA (G) and MB protein (H) expression after *Pgc1a* or *Nrf1* knockdown in differentiated imBA cells. Gene expression is relative to control cells and normalized to *36b4* expression. Protein expression is relative to control cells and normalized to ACTB expression (*n* = 3/3/3). Data are presented as mean ± SEM of at least two or three independent experiments, if not stated otherwise. Statistical significance was evaluated by one‐way ANOVA with Dunnett's post hoc test (A–C, H), uncorrected multiple *t*‐tests (D), or by two‐way ANOVA with Šídák's post hoc (E,F) or Tukey's (G) post hoc. *p‐value < .05, **p‐value < .01, ***p‐value < .001.

Next, we aimed to investigate gene regulatory mechanisms that control the temperature‐dependent expression of *Mb* in BAT, which correlated with *Ucp1* expression in vivo (Figure 1D). *Ucp1* expression in BAT is mainly driven by adrenergic activation of the brown adipocytes,^5^ but also activation via the cold‐sensing transient receptor potential melastatin 8 (TRPM8)^30^ and cell‐autonomous mechanisms^31^ have been reported. We, therefore, investigated the regulatory effects of adrenergic agonists and temperature on *Mb* expression in imBA cells in vitro. While norepinephrine (NE) and the β_3_‐adrenergic agonist CL316,243 (CL) highly increased *Ucp1* expression in differentiated imBA (Figure 2A), they significantly decreased *Mb* mRNA and protein levels by ∼50% (Figure 2B,C). This was also confirmed in primary brown adipocytes (Figure S2A). In vivo though, chronic CL‐treatment for 10 days increased *Mb* expression in iWAT and eWAT of male C57Bl/6N mice, without additionally increasing *Mb* expression in BAT (Figure 2D). Also, culturing imBAs at lower temperatures (30°C) did not affect *Mb* mRNA expression, nor did activation of TRPM8 by menthol (Figure S2B,C). As *Mb* expression was significantly induced during adipogenesis (Figure 1F), we investigated the role of PPARG in the regulation of *Mb* expression, treating differentiated adipocytes with PPARG‐agonist rosiglitazone (Figure S2D) or various concentrations of fatty acids (FA) (linoleic or oleic acid, Figure S2E,F). Both stimuli did not additionally increase *Mb* gene expression in vitro.

We further investigated *Mb* promotor methylation at a region comprising four CpG sites that constitute a putative nuclear respiratory factor 1 (NRF1) transcription factor binding site in BAT of C57BL/6N mice (housed at 30°C or 8°C, Figure S3). NRF transcription factors are activated by PGC1a, the key regulator of BAT thermogenesis, and control expression of mitochondrial proteins and other factors that regulate respiration.^32^ Methylation levels at that specified region consisting of four CpG sites was high (on average ∼88%) and there was a significant temperature effect on overall promoter methylation at the region, which was reduced in BAT of cold‐exposed mice (*p < .05 for temperature effect), without reaching significance at individual sites (Figure 2E). To confirm the role of PGC1a and NRF1 in the regulation of *Mb* expression in brown adipocytes, we knocked down *Pgc1a* or *Nrf1* in differentiated imBA adipocytes using siRNA (Figure 2F). While a reduction of *Pgc1a* expression by ∼50% resulted in significantly lower *Mb* gene and protein expression (by ∼50% compared to control transfected imBA, Figure 2G,H), particularly knockdown of *Nrf1* (by ∼70%) reduced MB protein expression by almost 90% (Figure 2G,H). These data clearly support the important regulatory role of both transcription factors in MB expression in brown adipocytes, with cold‐induced epigenetic changes potentially enhancing Nrf1 binding.

### 2.3 Reduced MB expression in brown adipocytes limits responsiveness to forskolin, lipolysis and mitochondrial respiration

**FIGURE 3:**
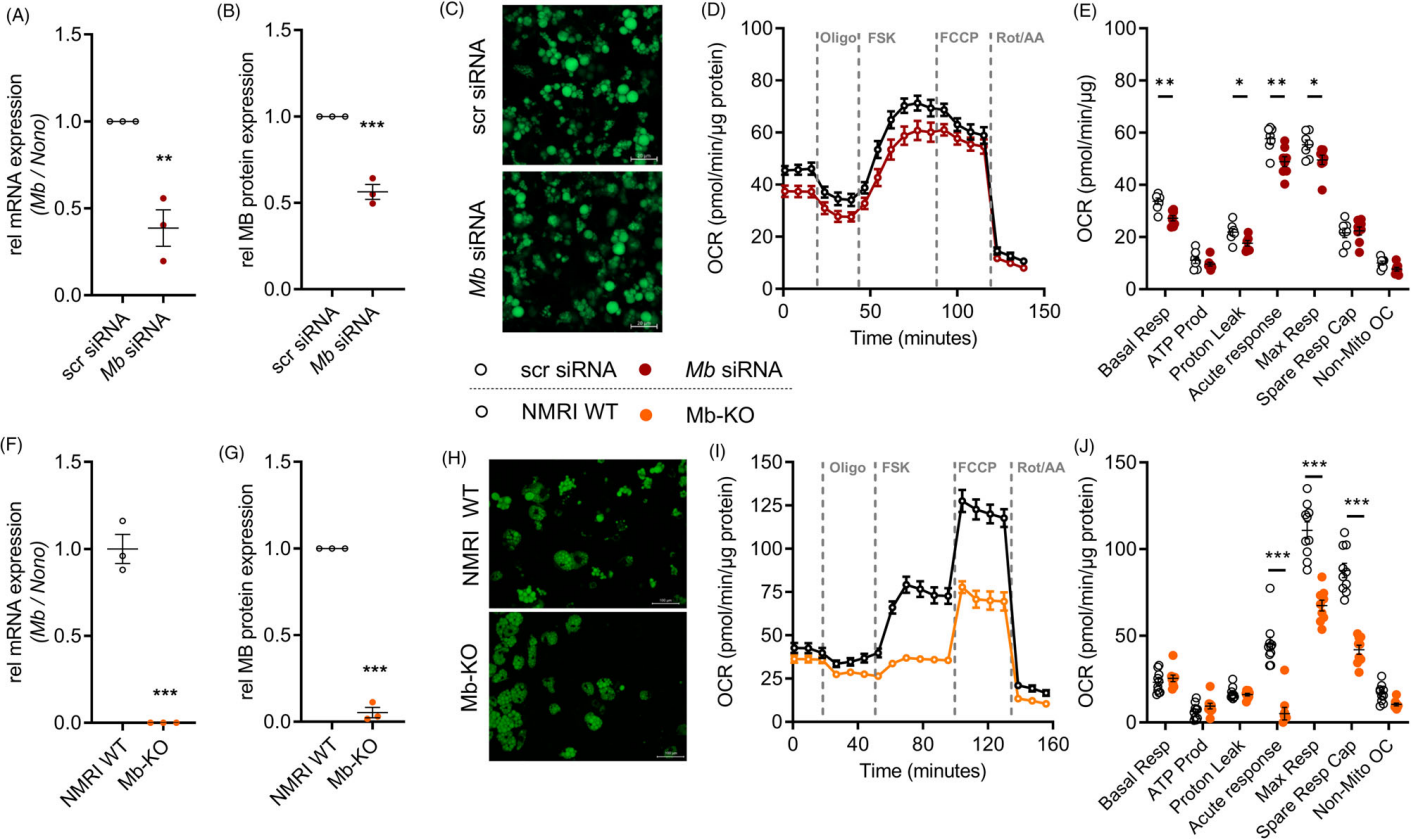
*Mb*‐knockdown in imBA or *Mb* knockout in primary brown adipocytes limits mitochondrial respiratory capacity and acute response to adrenergic activation. Reverse transfection with *Mb*‐siRNA significantly reduces MB mRNA (A) and protein (B) expression by ∼50% in differentiated imBA cells. (C) Fluorescence microscopy of AdipoRed‐stained lipid droplets in differentiated scrambled (scr) and *Mb*‐siRNA transfected imBA adipocytes (day 8). (D) Time‐resolved OCR of scr‐ and *Mb*‐siRNA transfected imBA measured by Seahorse (representative experiment, *n* = 6/8). (E) Quantification of basal respiration, ATP production, proton leak, acute response to forskolin (FSK), maximum and spare respiratory capacity and non‐mitochondrial respiration of samples in panel (D). *Mb* mRNA (F) and MB protein (G) expression in differentiated primary brown adipocytes from male NMRI WT and Mb‐KO mice. (H) Fluorescence microscopy of AdipoRed‐stained lipid droplets in differentiated primary brown adipocytes from male NMRI WT and Mb‐KO mice (day 8). (I) Time‐resolved OCR of differentiated primary brown adipocytes from male NMRI WT and Mb‐KO mice measured by Seahorse (representative experiment, *n* = 9/10). (J) Quantification of basal respiration, ATP production, proton leak, acute response to FSK, maximum and spare respiratory capacity and non‐mitochondrial respiration of samples in panel I. Data are presented as mean ± SEM of at least three independent experiments, if not stated otherwise. Statistical significance was evaluated by unpaired *t*‐tests (A,B,F,G), with multiple unpaired *t*‐tests corrected by the Holm–Šídák method (E,J). *p‐value < .05, **p‐value < .01, ***p‐value < .001. Scale bar: 20 μm (C), 100 μm (H).

To examine the consequences of reduced or lack of MB expression on energy metabolism in brown adipocytes, we used siRNA‐mediated knockdown of *Mb* in imBA as well as primary brown adipocytes from male Mb‐KO mice that were differentiated in vitro. Reverse transfection with siRNA resulted in a significant reduction of MB mRNA and protein levels by ∼50% in differentiated imBA compared to control cells (Figure 3A,B). We did not observe changes in lipid droplet size in both differentiated imBA after the knockdown of MB and primary brown adipocytes from Mb‐KO mice, compared to controls (Figure 3C,H).

Real‐time oxygen consumption rate (OCR) analysis in *Mb*‐knockdown imBA revealed a lower OCR in general (Figure 3D) with significant reductions in maximal respiration (Figure 3E). Also, MB‐knockdown in imBA reduced respiratory response to forskolin (FSK) compared to control adipocytes (∼15%, Figure 3D).

In differentiated primary brown adipocytes of Mb‐KO mice, MB mRNA and protein expression were not detectable (Figure 3F,G). In line with the results of the *Mb*‐knockdown in imBA, maximal respiration and spare capacity were reduced by ∼40%‐50% in *Mb*‐deficient adipocytes, without significantly affecting proton leak (Figure 3I,J). Moreover, brown adipocytes from Mb‐KO mice showed only a very limited response to FSK compared to control adipocytes (∼15%). Similar results were obtained using differentiated primary brown adipocytes from female Mb‐KO and NMRI WT mice (Figure S1D,E). These data provide the first evidence that MB exerts a functional role in BAT and show that MB expression levels control mitochondrial respiratory capacity and responsiveness to adrenergic signalling and lipolysis.

### 2.4 Increased MB expression in brown adipocytes results in smaller lipid droplets, increased responsiveness to adrenergic activation, lipolysis and mitochondrial respiration

**FIGURE 4:**
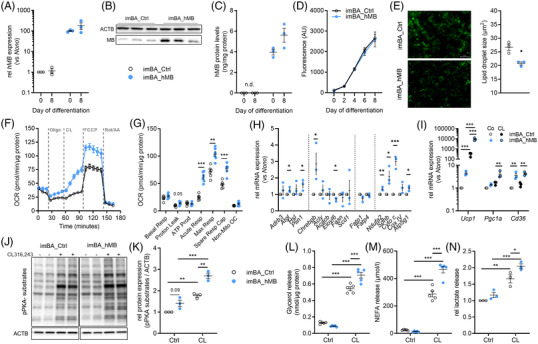
Overexpression of MB in imBA cells increases mitochondrial respiration and acute response to adrenergic stimulation. (A) Expression of *hMB* in stably transfected imBA_Ctrl and imBA_hMB adipocytes at day 0 and day 8 of differentiation. Gene expression is relative to imBA_Ctrl at day 0 and normalized to *Nono*. (B) Western blot analysis of total (mouse and human) MB expression and (C) ELISA‐based quantification of hMB protein in differentiated imBA_Ctrl and imBA_hMB adipocytes. (D) Quantification of lipid accumulation in imBA_Ctrl and imBA_hMB adipocytes during differentiation. (E) Fluorescence microscopy (left panel) and droplet size measurements (right panel) of AdipoRed‐stained lipid droplets in differentiated imBA_Ctrl and imBA_hMB adipocytes (day 8). (F) Time‐resolved OCR of differentiated imBA_Ctrl and imBA_hMB adipocytes measured by Seahorse (representative experiment, *n* = 6/6). (G) Quantification of basal respiration, proton leak, ATP production, acute response to CL316,243 (CL), maximum and spare respiratory capacity and non‐mitochondrial respiration of samples in panel F. (H) Lipolytic (*Adrb3, Atgl, Hsl, Plin1*), lipogenic (*Chrebpb, Acly, Acacb, Elovl6, Fasn, Scd1*), fatty acid (FA) transporter (*Fatp1, Fatp4*), and mitochondrial (*Ndufa2, Sdhb, Cycs, CoxVI, Atp5g1*) gene expression in differentiated imBA_Ctrl and imBA_hMB adipocytes. (I) Basal and CL‐induced thermogenic (*Ucp1, Pgc1a, Cd36*) gene expression in differentiated imBA_Ctrl and imBA_hMB adipocytes. (J) Western blot analysis and (K) quantification of basal and CL‐induced PKA‐activation in differentiated imBA_Ctrl and imBA_hMB adipocytes. Upper panel: anti‐phospho‐PKA substrate antibody; lower panel: anti‐ACTB antibody. (L) Measurements of basal and CL‐induced glycerol, (M) free fatty acid (free FA) and (N) lactate release from differentiated imBA_Ctrl and imBA_hMB adipocytes. Data are presented as mean ± SEM of at least two (L,M) or three independent experiments. Statistical significance was evaluated by two‐way ANOVA with Šídák's (A,C) or Tukey's (I–N) post hoc test or unpaired *t*‐test (E), or multiple unpaired *t*‐tests corrected by the Holm–Šídák method (G) or uncorrected (H). *p‐value < .05, **p‐value < .01, ***p‐value < .001. Scale bar: 50 μm.

Next, we evaluated whether overexpression of MB results in the opposite effect and improves mitochondrial respiration in imBA cells. Therefore, we generated human MB‐overexpressing imBA cells (imBA_hMB) and corresponding empty‐vector transfected controls (imBA_Ctrl) to investigate the effects of increasing MB expression on mitochondrial respiration and adrenergic activation of adipocyte lipolysis. ImBA_hMB cells exhibited high expression of hMB, as demonstrated by qPCR, Western blot and ELISA (Figure 4A–C). Overexpression was estimated to be ∼3‐5 fold compared to imBA_Ctrl cells, based on Western blot and total MB ELISA data (∼80 ng/mg total protein (imBA and imBA_Ctrl) compared to ∼290 ng/mg total protein). Both imBA cell lines showed similar proliferation (Figure S4A) and differentiation capacity into mature adipocytes with highly increased lipid incorporation after induction of adipogenesis (Figure 4D). Marker genes of adipogenesis, such as *Cebpa, Cebpb, Pparg* and *Adipoq*, were comparably induced in both clones (Figure S4B). While quantification of lipid incorporation did not show any differences, fluorescence microscopy and subsequent determination of lipid droplet size revealed significantly smaller droplets in imBA_hMB cells compared to controls (Figure 4E).

Measurement of OCR in imBA_hMB adipocytes demonstrated a significant increase in proton leak and acute response to adrenergic activation using β_3_‐adrenergic receptor agonist CL, as well as maximal respiration and spare mitochondrial capacity compared to control cells (Figure 4F,G). Similarly, the acute respiratory response to FSK was significantly higher in imBA_hMB compared to controls (Figure S4C). Expression of lipolytic genes was higher for *Atgl* and *Plin1* (no significant differences for *Adrb3* and *Hsl;* Figure 4H), and also lipogenic transcription factor *Chrebpb* was significantly increased, but this did not translate in altered expression of de novo lipogenesis enzymes (*Fasn, Acly, Acacb, Elovl6* and *Scd1*; Figure 4H). The expression of fatty acid transporters *Fatp1* and *Fatp4* were not different (Figure 4H). ImBA_hMB mitochondrial content was not significantly different from controls (Figure S4D), but imBA_hMB showed higher expression of mitochondrial OXPHOS genes (*NduFa2* (complex I), *Sdhb* (complex II), *Cycs* (complex III), *Atp5g1* (complex V); Figure 4H) and proteins SDHA, COXIV and CYCS (Figure S4E,F). Expression and membrane localization of ADRB3 was not different between imBA_hMB and controls (Figure S4G). Furthermore, the expression of thermogenic genes *Ucp1*, *Cd36* and *Pgc1a* were higher in imBA_hMB and this was significant after CL treatment (Figure 4I).

This translated into increased adrenergic responsiveness in imBA_hMB. Phosphorylation of protein kinase A (PKA) substrates was significantly higher in imBA_hMB adipocytes after CL treatment compared to controls (Figure 4J,K). While glycerol and non‐esterified FA release from imBA_hMB were comparable to controls in the basal state, they were significantly higher after CL stimulation (Figure 4L,M). CL‐induced lactate release was significantly higher in imBA_hMB compared to controls (Figure 4N) and increased metabolism was also reflected by more rapid acidification of cell culture medium with imBA_hMB clones under standard culturing conditions (Figure S4H).

Overall, these in vitro data clearly demonstrate the important function of MB in brown adipocytes by increasing mitochondrial respiration and capacity, as well as responsiveness to adrenergic activation of lipolysis.

### 2.5 MB is in part localized in brown adipocyte mitochondria and MB fatty‐acid binding is essential to increase mitochondrial capacity without affecting ROS

**FIGURE 5:**
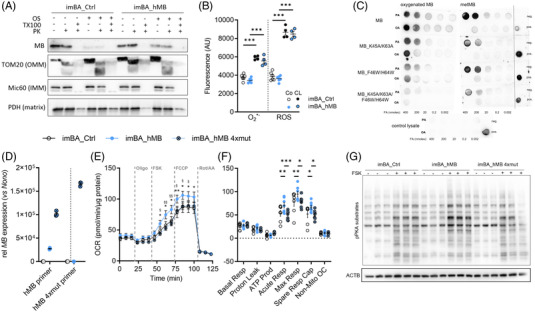
Mitochondrial localization of brown adipocyte MB and testing of non‐lipid binding MB mutant. (A) Western blot analysis of mitochondrial protease protection assay on isolated mitochondria of differentiated imBA_Ctrl and imBA_hMB adipocytes treated with osmotic shock (OS), Triton‐X‐100 (TX100) and/or protease K (PK). Upper to lower panels: anti‐MB, anti‐translocase of outer mitochondrial membrane (TOM) 20, anti‐MICOS complex subunit Mic60 (Mic60/Mitofilin) and anti‐pyruvate dehydrogenase (PDH) antibody. IMM/OMM: inner/outer mitochondrial membrane. (B) Quantification of intracellular superoxide (O_2_^−^) and reactive oxygen species (ROS) in differentiated imBA_Ctrl and imBA_hMB adipocytes under basal conditions and after CL stimulation (*n* = 6/4). (C) Dot blot lipid overlay assay of oxygenated (oxyMB) and further oxidized metmyoglobin (metMB) and mutants. Binding was detected using an anti‐MB antibody. Palmitic (PA) and oleic acid (OA) were spotted in indicated quantities. Respective MB proteins served as positive (pos) controls and solvent as negative (neg) control. (D) mRNA expression of *MB* and *hMB* 4xmutant in differentiated imBA_Ctrl, imBA_hMB and imBA_hMB 4xmutant (4xmut) clones using *MB* wildtype and mutant‐specific primers. Gene expression is relative to imBA_Ctrl and normalized to *Nono*. (E) Time‐resolved OCR of differentiated primary brown adipocytes expressing wildtype MB, non‐lipid binding MB (4xmut) or controls measured by Seahorse (representative experiment, *n* = 7/7/7). (F) Quantification of basal respiration, proton leak, ATP production, acute response to FSK, maximum and spare respiratory capacity and non‐mitochondrial respiration of samples in panel E. (G) Western blot analysis of PKA‐activation by FSK in differentiated control, hMB and hMB 4xmut imBA. Upper panel: anti‐phospho‐PKA substrate antibody; lower panel: anti‐ACTB antibody. Data are presented as representative or mean ± SEM of two or three independent experiments. Statistical significance was evaluated by one‐way ANOVA with Šídák's (B) or two‐way ANOVA with Tukey's (F) post hoc test. *p‐value < .05, **p‐value < .01, ***p‐value < .001.

Previous studies have demonstrated mitochondrial localization of MB in skeletal muscle^18^, ^19^ and also mesenchymal stem cells.^33^ We first analyzed whether MB is also localized in isolated mitochondria from imBA_hMB and controls. Most of MB was found in the cytosolic fraction, but MB was also detected in the crude mitochondrial fraction (Figure S4I). To analyze the mitochondrial integration of MB in imBA clones and controls, we performed a mitochondrial protease protection assay using protease K (PK). In agreement with previous studies, these experiments showed that the majority of mitochondrial MB is localized on the outer mitochondrial membrane (OMM, Figure 5A). Significant levels of MB remained after PK digestion of OMM proteins, which was not detectable anymore after Triton‐X‐100 (TX100) treatment. This indicated that part of MB is also localized within the mitochondria. To analyze the potential localization of MB at the inner mitochondrial membrane (IMM), we used osmotic shock (OS) treatment to disrupt the OMM and isolate the mitoplasts (Figure 5A). After OS treatment, MB was still detectable but reduced compared to whole mitochondria as OMM‐localized MB is removed from this sample. PK treatment of isolated mitoplasts did not reduce MB levels, which again was gone with the addition of TX100 and disintegration of the IMM/mitoplasts. Together, these results indicate that in brown adipocytes a significant part of mitochondrial MB is localized on the matrix side of the IMM. Mitochondrial localization of MB may protect against or regulate ROS generated during periods of increased mitochondrial respiratory chain activity. We performed ROS assays in imBA_hMB cells and controls. These data revealed no effects of MB overexpression on superoxide and ROS species in general under basal and CL‐treated conditions (Figure 5B).

We next investigated, whether MB's lipid binding property may underlie the observed beneficial effect on brown adipocyte metabolism. We, therefore, generated mutant non‐lipid‐binding MB based on results from molecular dynamic simulations.^34^ The mutations were predicted to affect interactions of MB with the acid group of the fatty acid (K45A and K63A) and the alkyl chain via the “gate‐keeping” residues (F46W and H64W).^34^ Lipid binding of FAs oleate and palmitate by MB and mutants were then assessed by dot blot lipid overlay assays (Figure 5C). These clearly confirmed the binding of oxygen‐carrying MB (oxyMB) and the further oxidized metmyoglobin (metMB) to both FAs. Importantly, MB mutants demonstrated reduced binding for each double mutant (MB_ K45A/K63A and MB_F46W/H64W) and almost complete loss of binding for the mutant lacking all four residues (MB_K45A/F46W/K63A/H64W). We then generated imBA clones overexpressing the non‐lipid binding MB mutant (imBA_hMB 4xmut; Figure 5D). All imBA clones showed comparable differentiation potential and mitochondrial content to control imBA cells (Figure S4D,J), with the mutant MB being slightly higher expressed than the wildtype (14 vs. 3.3 ng/mg total protein). Finally, measurement of OCR in the differentiated imBA clones demonstrated that mutation of the lipid‐interacting residues in human MB fully abolished the beneficial effect of MB overexpression in brown adipocytes (Figure 5E,F). Also, the enhancement of PKA activation was lost when mutating FA‐interacting residues of MB and reached levels comparable to CL‐stimulated imBA_Ctrl adipocytes (Figure 5G).

Together, these data indicate lipid binding as the important property of brown adipocyte MB to enhance substrate flux and enable increased mitochondrial respiration and thermogenesis. MB‐overexpressing brown adipocytes further seem to adapt to this situation with increased mitochondrial and thermogenic gene and protein expression.

We next asked whether MB expression had similar effects in white adipocytes with potential consequences for WAT browning.

### 2.6 Increased MB expression in white adipocytes results in increased responsiveness to adrenergic activation, lipolysis and mitochondrial respiration

**FIGURE 6:**
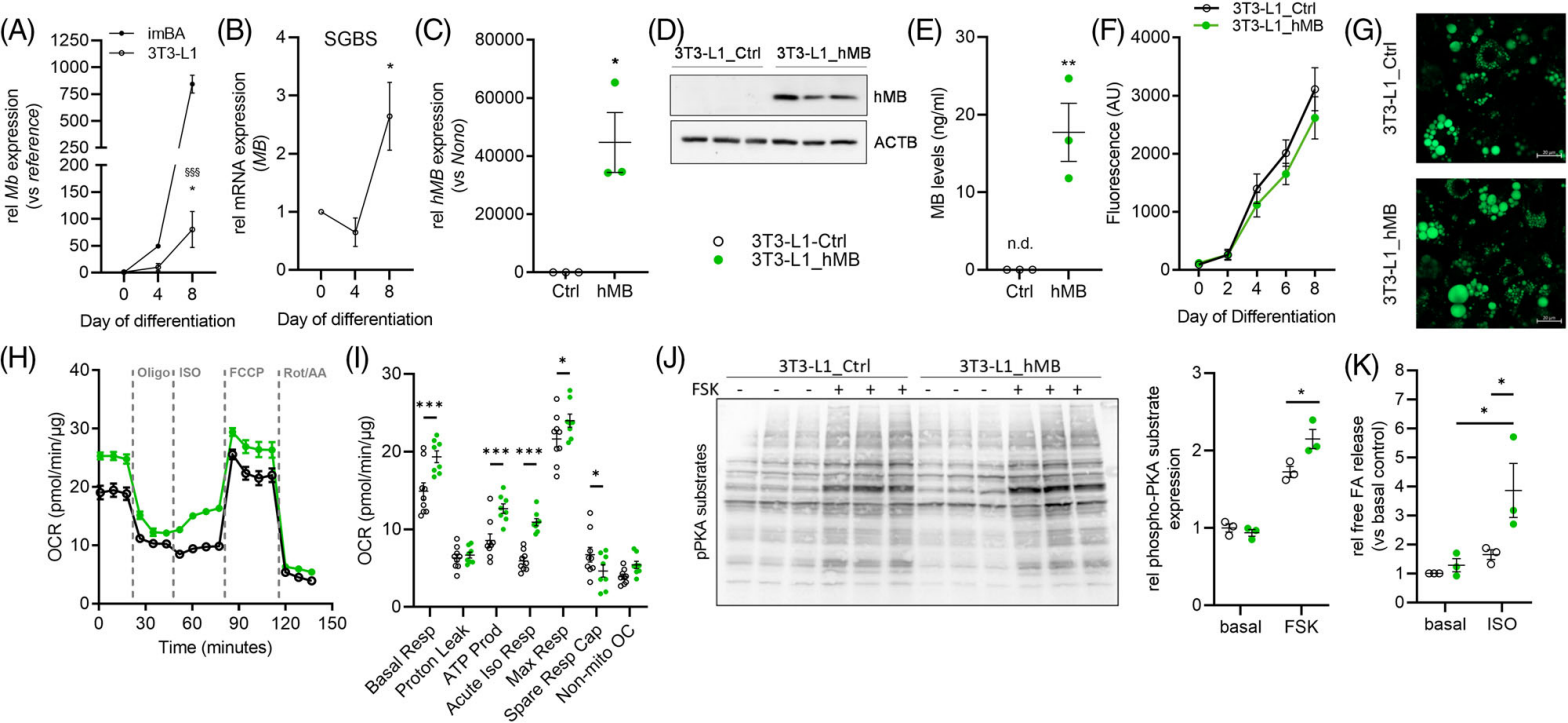
MB expression is increased during differentiation and increases mitochondrial respiration in white adipocytes. (A) *Mb* mRNA expression in mouse 3T3‐L1 (compared to imBA data as of Figure 1F; * vs day 0, §§§ vs imBA day 8) and (B) human SGBS cells during differentiation, relative to day 0. (C) Expression of *hMB* mRNA as well as (D) Western blot analysis and (E) ELISA‐based quantification of MB protein expression in transfected and differentiated 3T3‐L1_Ctrl and 3T3‐L1_hMB adipocytes. Gene expression is relative to imBA_Ctrl and normalized to *Nono*. (F) Quantification of lipid accumulation in 3T3‐L1_Ctrl and 3T3‐L1_hMB adipocytes during differentiation. (G) Fluorescence microscopy of AdipoRed‐stained lipid droplets in differentiated 3T3‐L1_Ctrl and 3T3‐L1_hMB adipocytes. (H) Time‐resolved OCR of differentiated 3T3‐L1_Ctrl and 3T3‐L1_hMB adipocytes measured by Seahorse (representative experiment, *n* = 9/8). (I) Quantification of basal respiration, proton leak, ATP production, acute response to isoproterenol (ISO), maximum and spare respiratory capacity and non‐mitochondrial respiration of samples in panel (H). (J) Western blot analysis and quantification of basal and FSK‐induced PKA‐activation in differentiated 3T3‐L1_Ctrl and 3T3‐L1_hMB adipocytes. (K) Measurements of basal and ISO‐induced free fatty acid (FA) release from differentiated 3T3‐L1_Ctrl and 3T3‐L1_hMB adipocytes. Data are presented as mean ± SEM of at least two independent experiments, if not stated otherwise. Statistical significance was evaluated through two‐way ANOVA with Fisher's LSD test (A,I), one‐way ANOVA with Dunnett's post hoc test (B), unpaired *t*‐tests (C,E), or two‐way ANOVA with Šídák's (J) or Tukey's (K) post hoc test. *p‐value < .05, **p‐value < .01, ^§§§^/***p‐value < .001. Scale bar: 20 μm.

Expression of *Mb* in white adipocytes was analyzed using murine 3T3‐L1 as well as human SGBS adipocytes. In both cell lines, *Mb* expression was significantly induced during differentiation (Figure 6A,B). Induction of *Mb* in white 3T3‐L1 was ∼10× lower than in brown imBA cells (Figure 6A). To investigate, whether increased MB expression in white adipocytes also increases metabolic activity, we overexpressed human MB in 3T3‐L1 adipocytes (3T3‐L1_hMB) as demonstrated by qPCR, Western blot and ELISA (Figure 6C–E). Lipid incorporation and droplet size (Figure 6F,G) and gene expression related to adipogenesis (*Pparg, Adipoq, Cebpa*), lipid uptake and metabolism (*Fabp4, Cd36, Fasn*) were highly and comparably induced during differentiation in 3T3‐L1_hMB and 3T3‐L1_Ctrl adipocytes (Figure S5A–B). 3T3‐L1_hMB cells exhibited significantly increased basal and maximal mitochondrial respiration with higher ATP production compared to controls (3T3‐L1_Ctrl), but no difference in proton leak (Figure 6H,I). Furthermore, 3T3‐L1_hMB adipocytes showed a significant acute respiratory response to isoproterenol (ISO) compared to controls (Figure 6H,I), and also adrenergic induction of PKA substrate‐phosphorylation and free FA release was significantly higher (Figure 6J,K). Yet, lipid droplet size was not affected by MB overexpression (data not shown) and also thermogenic gene (*Ucp1, Adrb3* and *Cidea*), as well as mitochondrial OXPHOS protein expression, was not different (Figure S5C,D). Gene expression of *Ryr2* and *Serca2b*, central genes of Ca^2+^‐cycling based UCP1‐independent thermogenesis in beige AT,^35^ were not changed in 3T3‐L1 clones (Figure S5E). Together, these data clearly show that white adipocytes featuring increased MB expression exhibit improved respiratory capacity and adrenergic sensitivity, and this could be an important determinant in beige adipocyte thermogenesis or adipocyte browning.

### 2.7 Mb‐KO mice show impaired thermoregulation at temperatures below thermoneutrality

**FIGURE 7:**
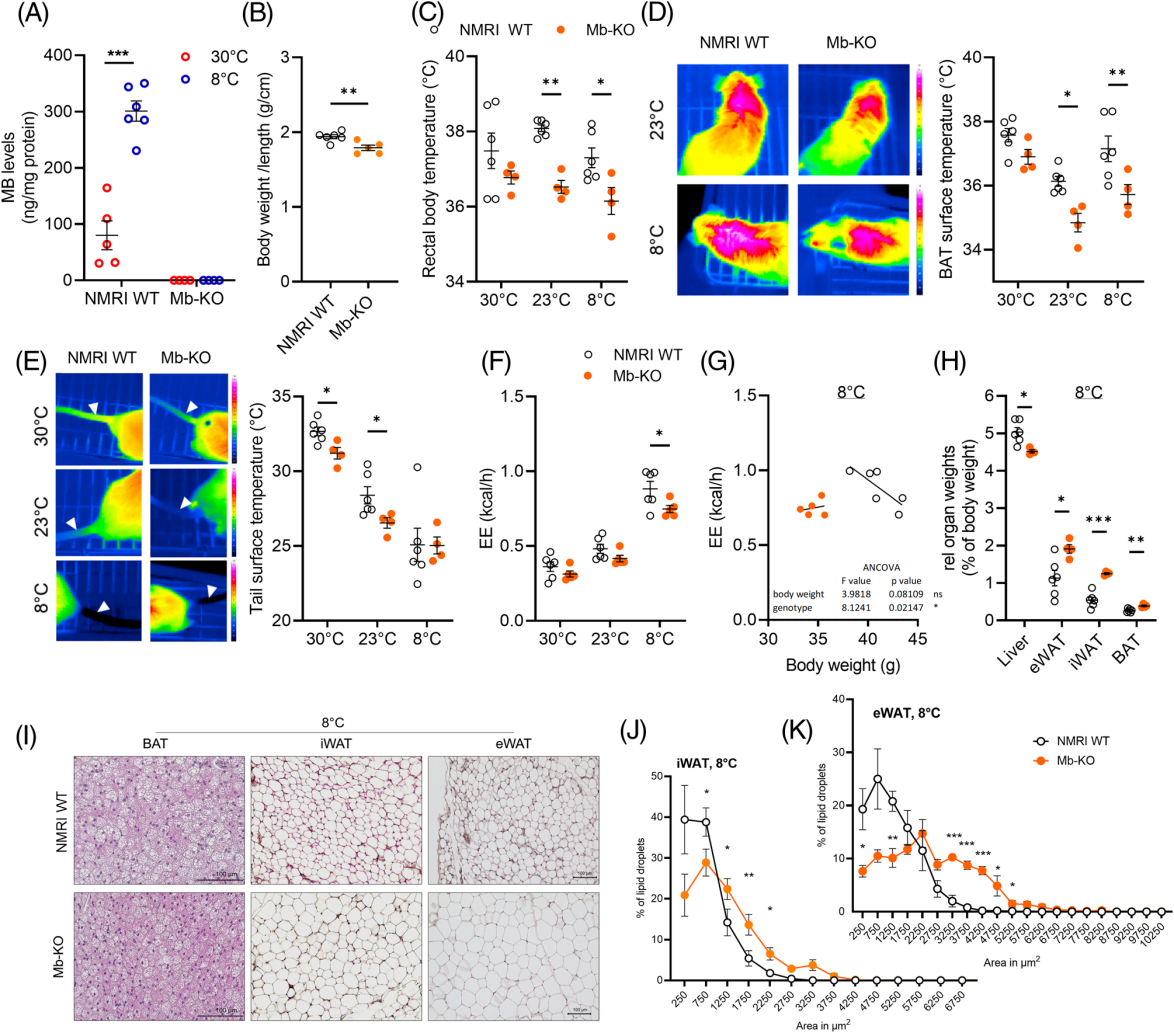
Mb‐KO mice show impaired thermoregulation at temperatures below thermoneutrality. (A) MB protein expression measured by ELISA in BAT lysates of male NMRI WT and Mb‐KO mice housed at 30°C or 8°C for 1 week (NMRI WT data as in Figure 1D). (B) Body weight per length of NMRI WT and Mb‐KO mice. (C) Rectal body, (D) BAT surface and (E) tail surface temperatures in NMRI WT and Mb‐KO mice housed at 30°C, 23°C and 8°C for 1 week (*n* = 6/4 per group). Thermal images from BAT and tails of NMRI WT and Mb‐KO mice are shown in D and E, respectively. (F) Energy expenditure (EE) in NMRI WT and Mb‐KO mice subsequently housed at 23°C, 30°C and 8°C during indirect calorimetry (*n* = 6/5 per group). (G) Regression plots of EE against body weight with the ANCOVA test using body weight as a covariate for mice housed at 8°C. (H) Relative organ weights of epididymal (eWAT), inguinal (iWAT) AT, BAT and liver of NMRI WT and Mb‐KO mice housed at 8°C for 1 week (*n* = 6/5 per group). (I) Representative H&E‐stained sections of BAT (40×), iWAT and eWAT (20×) from NMRI WT and Mb‐KO mice housed at 8°C. (J) Adipocyte lipid droplet size distribution in iWAT and (K) eWAT from NMRI WT and Mb‐KO mice shown in I (*n* = 5/4). Data are shown as mean ± SEM. Statistical significance was evaluated by two‐way ANOVA with Šídák's post hoc test, unpaired *t*‐tests (B), or uncorrected multiple *t*‐tests (J,K). *p‐value < .05, **p‐value < .01, ***p‐value < .001. Scale bar: 100 μm.

We next investigated thermoregulation in previously described whole‐body Mb‐KO mice. Animals were housed at different ambient temperatures for 7 days, either at thermoneutrality, at 23°C or 8°C. To validate the model, we measured BAT MB expression in Mb‐KO and NMRI WT mice housed at thermoneutrality and 8°C. BATs of NMRI WT mice showed a significant and 10‐fold increase in MB protein levels after cold exposure, while Mb‐KO BATs had no detectable MB expression (Figure 7A). In contrast to previous studies,^24^, ^27^ we observed that NMRI WT animals were significantly heavier compared to Mb‐KO of the same age (Figure 7B). After 5 days at the respective temperatures, we observed differences in body temperature (Figure 7C) as well as BAT and tail surface temperatures (Figure 7D,E) between the genotypes. Under thermoneutral conditions, there was a trend for lower body and BAT temperatures in Mb‐KO compared to NMRI WT, and these differences became significant in Mb‐KO held at sub‐thermoneutral temperatures (23°C and 8°C, Figure 7C,D). We further investigated whether heat dissipation via the tail was affected in Mb‐KO. Tail surface temperature, as a surrogate parameter for vasoconstriction/vasodilation, was lower in Mb‐KO housed at thermoneutrality and 23°C compared to NMRI WT, indicating a compensatory reduction of heat‐loss via the tail (Figure 7E). There were no differences in animals held at 8°C. We next performed indirect calorimetry for Mb‐KO and NMRI WT mice housed at all three ambient temperatures. EE inversely correlated with ambient temperature and was twice as high in animals housed at 8°C compared to thermoneutrality (Figure 7F, Figure S6A). Furthermore, at 8°C EE of Mb‐KO was significantly lower than in NMRI WT. Importantly, ANCOVA analysis to adjust for the differences in body weight revealed a significant genotype‐driven effect that was independent of body weight (Figure 7G, Figure S6B,C for 30°C and 23°C). Food intake (g per g body weight) and respiratory exchange ratio (RER) were not different, indicating no changes in food preference (Figure S6D,E). Notably, Mb‐KO mice were less active at temperatures below thermoneutrality, both with respect to locomotor activity (X + Y) and rearing (Z) potentially contributing to the lower EE in these mice (Figure S6F,G).

Relative AT weights were higher in Mb‐KO mice at all temperatures, with significant differences for the WAT and BAT (Figure 7H for 8°C, Figure S6H,I for 30°C and 23°C). Relative liver weights in contrast were significantly lower in Mb‐KO mice at 8°C. BAT histology showed no obvious differences between Mb‐KO and NMRI WT held at thermoneutrality and after cold exposure (8°C in Figure 7I, 23°C and 30°C in Figure S6J). White adipocytes from iWAT and eWAT of Mb‐KO mice were significantly larger at all temperatures (8°C in Figure 7J,K, 23°C and 30°C in Figure S6K,L).

**FIGURE 8:**
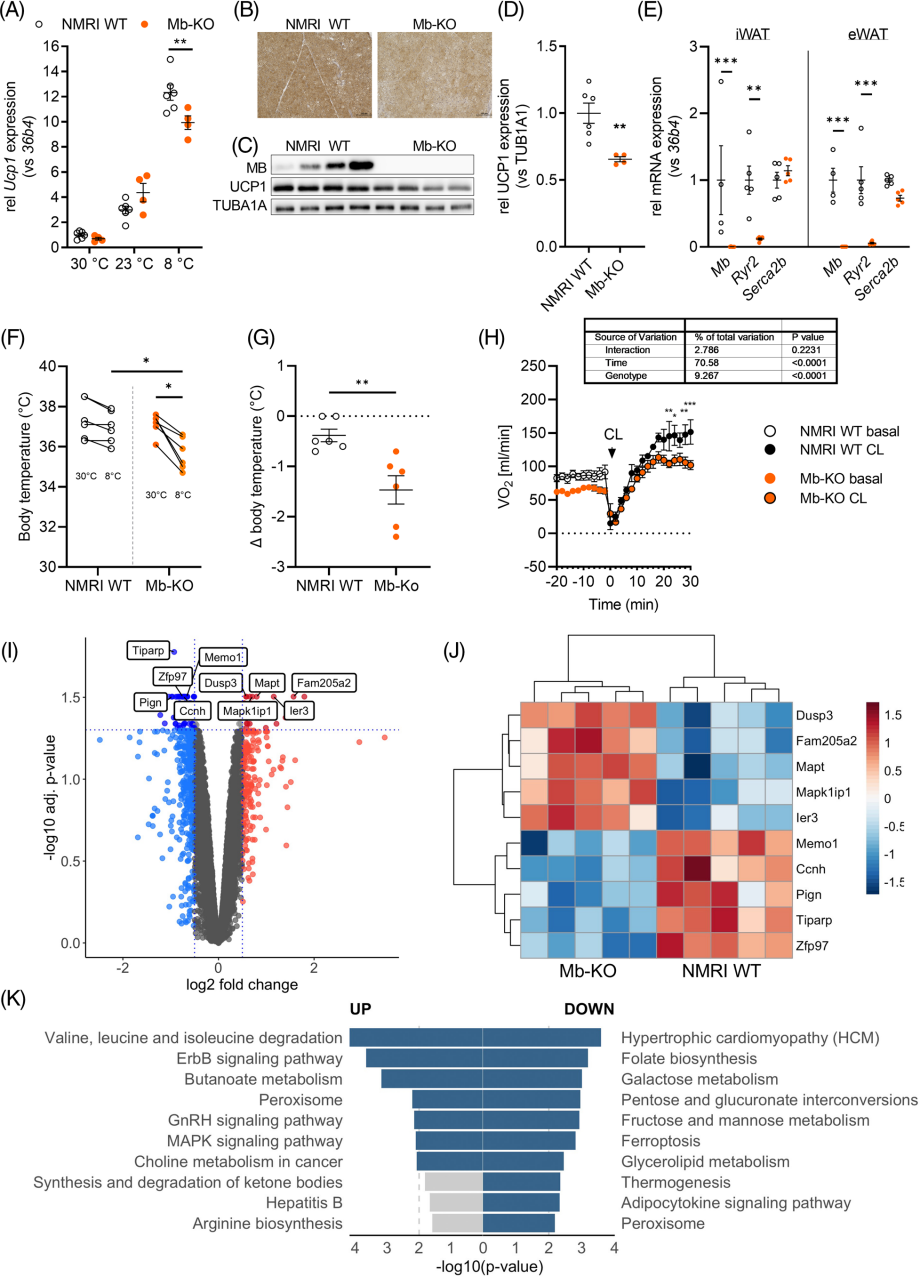
Differential gene expression in BAT of male NMRI WT and Mb‐KO mice at temperatures below thermoneutrality. (A) *Ucp1* mRNA expression in BAT of NMRI WT and Mb‐KO mice housed at 30°C, 23°C or 8°C for 1 week (*n* = 6/4). (B) Immunohistochemical stainings of UCP1 and (C) Western blot analysis of MB and UCP1 expression in BAT of cold‐exposed NMRI WT and Mb‐KO mice. Tubulin served as a loading control. (D) Quantification of UCP1 expression is shown in (C). (E) *Mb, Ryr2 and Serca2b* mRNA expression in iWAT and eWAT of NMRI WT and Mb‐KO mice housed at 23°C (*n* = 5/5). (F) Body temperature in male NMRI WT and Mb‐KO mice (*n* = 6/6) before and 6 h after the transition from thermoneutrality to 8°C and calculated changes in individual body temperatures (G). (H) Oxygen consumption rates (OCR) of male NMRI WT and Mb‐KO mice before and after a single i.p. injection of CL (*n* = 4/6). (I) Volcano plot of differentially expressed genes (DEG) in BAT of acutely cold‐exposed male NMRI WT and Mb‐KO mice. Microarray gene expression data in the volcano plot are displayed as log_2_ fold change (FC) versus the ‐log_10_ of the _adj_p value. Downregulated genes in Mb‐KO (_adj_p < .05 and log_2_ FC ≤ −0.5) compared to NMRI WT animals are shown in blue, whereas red colour encodes upregulated genes in Mb‐KO (_adj_p < .05 and log_2_ FC ≤ −0.5). Thresholds are shown as dashed lines. The top 10 DEGs are labelled with gene symbols. (J) Heatmap of top regulated genes in NMRI WT and Mb‐KO. Red and blue indicate genes higher or lower expressed in Mb‐KO compared to NMRI WT, respectively. (K) KEGG pathway functional enrichment analysis of DEGs from microarray partial analysis of genes related to mitochondrion and lipid metabolic processes selected based on GO terms (top ten functional pathways up‐ and downregulated). The vertical axis represents the KEGG pathway terms significantly enriched by the DEGs; the horizontal axis indicates ‐log_10_ (*p*‐value). Statistical significance was evaluated by multiple unpaired *t*‐tests corrected by the Holm‐Šídák method (A), unpaired *t*‐test (D), or two‐way ANOVA with Šídák's post hoc test (E). *p‐value <.05, **p‐value < .01. Scale bar: 100 μm.

Despite similar BAT morphology, *Ucp1* gene expression was significantly lower in BAT of Mb‐KO mice held at 8°C, as also indicated by UCP1 immunohistochemistry and Western blot analysis (Figure 8A–D). Expression of other thermogenic and mitochondrial genes (such as *Cidea, Cycs, CoxIV, Atg5g1, Fasn*) and OXPHOS complexes were not different (Figure S7A,B).

Yet, cold‐induced PKA activation and HSL phosphorylation were reduced in BAT of Mb‐KO mice (Figure S7C–E). We also analyzed gene expression of key players in beige AT Ca^2+^‐cycling, *Ryr2* and *Serca2b*, in eWAT and iWAT depots of cold‐exposed NMRI WT and Mb‐KO mice (Figure 8E). In support of a functional link between *Mb* expression and beige AT thermogenesis, we found a significant reduction (>90%) of *Ryr2* expression in both WAT depots of cold‐exposed Mb‐KO mice. *Serca2b* expression was not different between the genotypes.

In line with these findings, Mb‐KO mice showed impaired adaption to cold, exhibiting a significant drop in body temperature 6 h after the transition from thermoneutrality to 8°C (Figure 8F,G). To selectively study non‐shivering thermogenesis in brown and/or beige adipocytes, we measured oxygen consumption in response to an acute CL injection in male NMRI WT and Mb‐KO mice using indirect calorimetry. As expected, CL significantly increased oxygen consumption albeit to a significantly lower extent in Mb‐KO mice, as compared to NMRI WT mice (Figure 8H, Figure S7F,G). ANCOVA analysis to take account for the differences in body weight showed a significant genotype‐driven effect that again was independent of body weight (Figure S7F,G).

To get a broader insight into changes in BAT of Mb‐KO mice, we analyzed gene expression in BAT after acute cold exposure in male Mb‐KO and NMRI WT mice using microarrays. A total of 110 genes were significantly differentially expressed in Mb‐KO BAT compared to NMRI WT (31 genes upregulated, 79 genes downregulated in Mb‐KO BAT; _adj_ *p* < 0.05, |log_2_ FC| ≥ 0.5; Figure 8I; Table S3). The top five upregulated genes included *Dusp3*, *Mapt*, *Mapk1ip1, Fam205a2*, and *Ier3*, whereas *Tiparp, Zfp97, Memo1, Pign*, and *Ccnh* were the top five less abundantly expressed genes in Mb‐KO BAT (Figure 8I,J). Pathway and gene ontology analyses (Tables S5–S8) of higher expressed genes in BAT of Mb‐KO revealed enrichment of genes involved in MAPK signalling (_adj_ *p* = 0.0066; KEGG:04010; *Jun, Dusp3, Cacna1a, Mapt, Mapk8ip1*) and related to the cellular response to oxidative stress (_adj_ *p* = 0.0219; GO:0034599; *Jun, Mapt, Pdk2, Msra*). Less abundant genes were related to protein export (_adj_ *p* = 0.0273; KEGG:03060 *Srp54b, Srp54c, Srp54a*) and RNA binding (_adj_ *p* = 0.0031; GO:0003723; *Ptcd3, Ddx3y Ssb, Tfrc, Dhx9, Nucks1, Thoc2, Dhx40, Rsl1d1, Syncrip, Sumo2, G3bp2, Srsf3, Brix1, Naa15, Eif4e, Dcaf13, Lyar, Mrpl1*).

Focusing on microarray partial analysis of genes related to mitochondrion and lipid metabolic processes selected based on GO terms, 57 genes were differentially expressed in Mb‐KO BAT compared to NMRI WT (_adj_ *p* < 0.05, |log_2_ FC| ≥ 0.5; Table S4). Pathway analysis (Tables S9,S10) revealed that upregulated genes were related to branched‐chain amino acid degradation (KEGG:00280), ErbB signalling (KEGG:04012) and FA degradation (KEGG:00071), while less abundant genes were related to fructose, mannose and galactose metabolism (KEGG:00051, KEGG:00052) and thermogenesis (KEGG:04714) (Figure 8K).

Target genes *Ier3* (_adj_ *p* = 0.02, log_2_FC = 1.2), *Tfrc* (_adj_ *p* = 0.02, log_2_FC = ‐1.3), *Adcy10* (_adj_ *p* = 0.03, log_2_FC = ‐0.9), and *Igf1*(_adj_ *p* = 0.02, log_2_FC = ‐2) were selected from significant differentially regulated genes (DEGs) from the microarray subset analysis for validation by qPCR, whereas *Ier3* (_adj_ *p* = 0.03, log_2_FC = 1.2) and *Tfrc* (_adj_ *p* = 0.04, log_2_FC = ‐1.2) are also significant DEGs from the complete microarray study. Validation confirmed the increased *Ier3* and downregulated *Tfrc*, *Adcy10* and *Igf1* expression in Mb‐KO BAT after cold exposure (Figure S7H).

These results add novel insights into the interaction of MB and BAT metabolism and function. The loss of MB induces metabolic alterations in BAT that impair BAT activation and thermoregulation in Mb‐KO mice housed at temperatures below thermoneutrality or after treatment with adrenergic agonists. Furthermore, also UCP1‐independent beige AT thermogenesis seems to be affected by the loss of MB in white AT. Yet, in line with previous results, this did not translate into clear changes in whole‐body energy expenditure. It is important to note that Mb‐KO mice lack MB in all tissues, first of all, muscle, which in turn induces multiple compensatory mechanisms,^29^ which may obscure the effects of MB knockout in BAT on whole‐body energy expenditure.

### 2.8 *MB* expression in human AT is differentially regulated in subcutaneous and visceral AT and in obesity and correlates with UCP1 expression and other markers of AT browning

**FIGURE 9:**
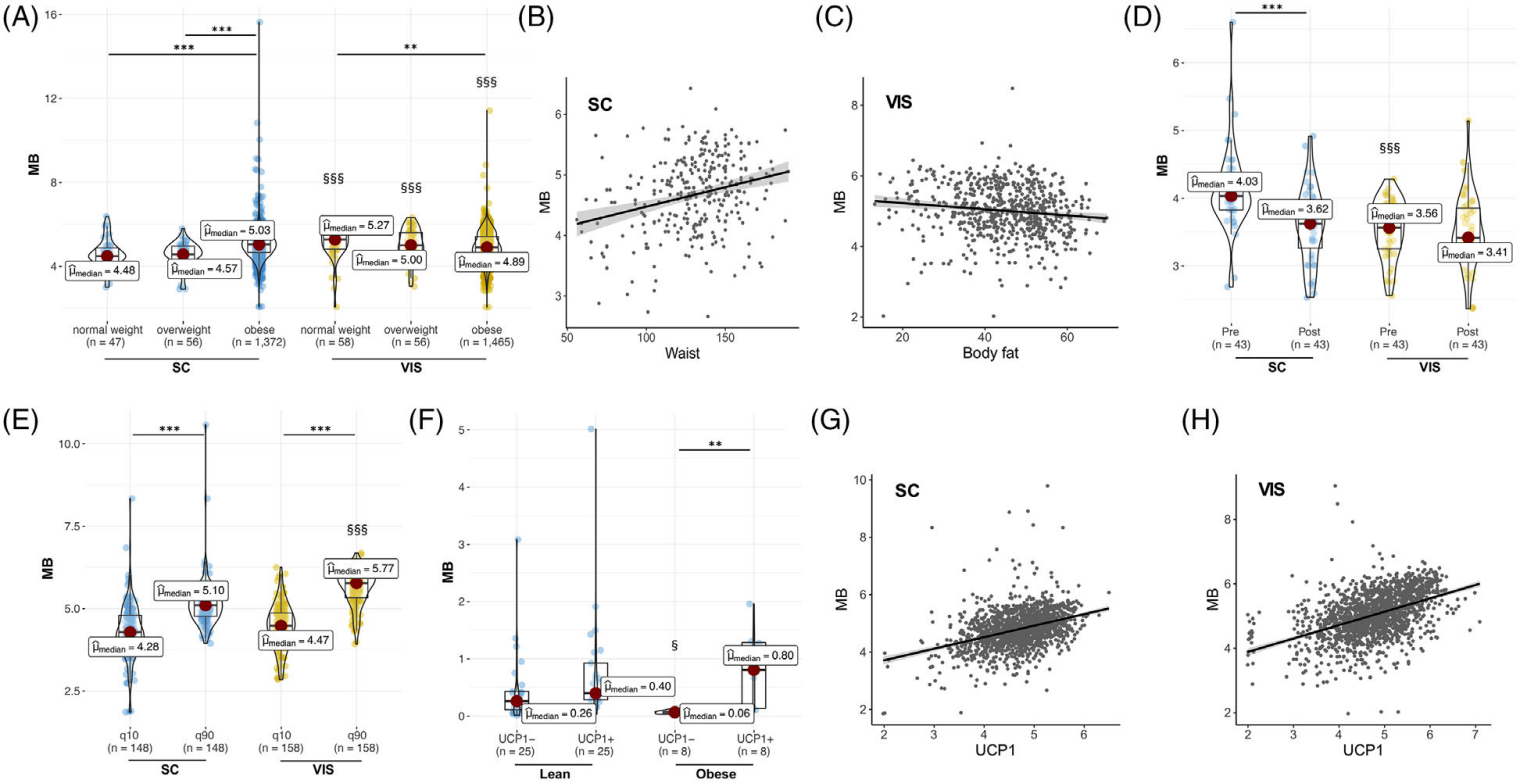
*MB* expression in human white adipose tissue. (A) Subcutaneous (SC) and visceral (VIS) adipose tissue *MB* gene expression in BMI subgroups (normal weight (20 ≤ BMI ≤ 25), overweight (25 < BMI < 30) and obese (BMI ≥ 30)). Adj. *p*‐values: SC normal weight versus SC obese (1.14 × 10^−5^), SC overweight versus SC obese (7.27 × 10^−7^), VIS normal weight versus VIS obese (1.06 × 10^−3^), SC normal weight versus VIS normal weight (7.56 × 10^−7^), SC overweight versus VIS overweight (1.14 × 10^−4^), and SC obese versus VIS obese (1.13 × 10^−4^). (B) Correlation of SC *MB* expression with waist circumference (*n*_pairs_ = 304; *ρ*_Spearman_ = 0.25; *p* = 1.19 × 10^−5^) and (C) inverse correlation of VIS *MB* expression with body fat (*n*_pairs_ = 648; *ρ*_Spearman_ = ‐0.17; *p* = 1.80 × 10^−5^) in humans. (D) *MB* gene expression in SC and VIS AT samples of the bariatric surgery cohort pre‐ and post‐surgery. (SC AT pre vs post: *p*‐value = 1.69 × 10^−4^; SC AT pre vs VIS AT pre *p*‐value = 2.16 × 10^−5^). (E) *MB* gene expression in SC and VIS of patient samples belonging either to the 10th quantile or 90th quantile of AT *UCP1* expression. Adj. *p*‐values: SC q10 versus SC q90 (2.21 × 10^−17^), VIS q10 versus VIS q90 (2.11 × 10^−40^), and SC q90 versus VIS q90 (1.19 × 10^−8^). (F) *MB* expression in SC of lean and obese children with or without detectable *UCP1* expression. *MB* gene expression was normalized to the mean of two reference genes (*ACTB* and *TBP*). Adj. *p*‐values: lean *UCP1* ^−^ versus obese *UCP1* ^−^ (0.03), and obese *UCP1* ^−^ versus obese *UCP1* ^+^ (4.92 × 10^−3^). (G) Correlation of *MB* expression in SC (*n*_pairs_ = 1473; *UCP1*: *ρ*_Spearman_ = 0.39; *p* = 1.26 × 10^−53^) and (H) VIS AT (*n*_pairs_ = 1578; *UCP1*: *ρ*_Spearman_ = 0.49; *p* = 3.72 × 10^−96^) with *UCP1*. Statistical significance was evaluated by Kruskal–Wallis one‐way ANOVA and Dunn's test for pairwise comparisons and corrected for multiple inference using the Holm method (A,D,E) or by Mann–Whitney U test (F). **p‐value < .01, ***p‐value < .001 (comparison between BMI subgroups (A,B) or UCP1‐expression quantile (E,F) within an AT depot), ^§^ *p*‐value < .05, ^§§§^ *p*‐value < .001 (comparison between AT depots within BMI subgroups (A) or pre‐ vs. post‐surgery samples (F) or UCP1‐expression quantile (E,F)).

Because human SGBS adipocytes showed an induction of *MB* expression during differentiation (Figure 6B), we finally analyzed *MB* expression in human WAT to address the clinical relevance of our data and potential association with parameters of obesity and/or AT browning in humans. We examined *MB* expression in subcutaneous (SC) and visceral (VIS) AT samples from a large human adult cross‐sectional cohort. In 3056 SC and VIS AT samples, we found differential *MB* expression with respect to the AT depots and the obesity state. In lean patients, *MB* expression was significantly lower in SC AT compared to VIS AT, whereas the opposite was found in patients with obesity (Figure 9A). When analysing subgroups of increasing BMI (from lean, overweight to obese patients), a significant and stepwise increase of SC *MB* expression was observed, and the opposite was found in VIS AT (Figure 9A). There were no sex‐specific differences in *MB* expression in VIS or SC AT depots, also when looking at BMI subgroups and diabetes state (Figure S8A). In SC AT, *MB* expression showed significant correlations to waist circumference (*n*_pairs_ = 304; *ρ*_Spearman_ = 0.25; *p* = 1.19 × 10^−5^), body weight (*n*_pairs_ = 1 442; *ρ*_Spearman_ = 0.12; *p* = 2.14 × 10^−6^) and BMI (*n*_pairs_ = 1,473; *ρ*_Spearman_ = 0.11; *p* = 1.05 × 10^−5^; Figure 9B and Figure S8B,C), whereas in VIS AT *MB* expression inversely correlated with body fat (*n*_pairs_ = 648; *ρ*_Spearman_ = −0.17; *p* = 1.80 × 10^−5^; Figure 9C). There were no significant correlations with parameters of dyslipidemia or insulin resistance (data not shown), but SC *MB* expression was inversely correlated with fT4 levels (*n*_pairs_ = 416; *ρ*_Spearman_ = −0.14; *p* = 0.004; Figure S8D). In the bariatric surgery cohort (average patient BMI > 50), we also found significantly higher *MB* expression in SC AT compared to VIS AT (*p* = 2.16 × 10^−5^; Figure 9D). Notably, we observed a significant reduction in SC AT *MB* expression (*p* = 1.69 × 10^−4^; Figure 9D) after weight loss which may indicate a reversal of the obesity‐driven *MB* expression after weight loss in SC AT. On the other hand, expression levels of *MB* in VIS AT may require further weight loss for a reversal toward higher expression observed in lean patients (Figure 9A), as post‐surgery patients still had a BMI > 40 post‐bariatric surgery.

Next, we investigated whether human AT *MB* expression may show associations with aspects of AT browning. First, we analyzed *MB* expression in AT samples with the highest (90^th^ quantile) or lowest (10^th^ quantile) *UCP1* expression (90^th^ and 10^th^ quantile each with *n* = 148 for SC and *n* = 158 for VIS; Figure 9E). Indeed, *MB* was significantly higher expressed in the SC (_adj_ *p* = 2.21 × 10^−17^) and VIS (_adj_ *p* = 2.11 × 10^−40^) samples belonging to the 90^th^ quantile of *UCP1* expression. Also, within the 90^th^ and 10^th^ quantile groups of *UCP1* expression, there were no AT depot‐specific differences in *MB* expression, also when sub‐dividing into lean and obese patients (Figure S8F). Significantly higher *MB* expression in *UCP1*‐positive AT samples was further confirmed in SC AT samples from 66 Caucasian children, both in lean and obese (*n* = 33 with detectable *UCP1* expression, *n* = 33 *UCP1*‐negative samples; Figure 9F). These data suggest, that higher *MB* expression is found in AT or adipocytes with a higher thermogenic potential. In line, *MB* expression in the adult cohort showed highly significant correlations not only with *UCP1* but also with additional markers of AT browning, including *TMEM26*, *UCP3* and *NRG4* ^36^, ^37^ in SC AT (*n*_pairs_ = 1 473; *UCP1: ρ*_Spearman_ = 0.39; *p* = 1.26 × 10^−53^, Figure 9G; *TMEM26: ρ*_Spearman_ = 0.21, *p* = 1.12 × 10^−15^; *UCP3: ρ*_Spearman_ = 0.51, *p* = 1.35 × 10^−98^; *NRG4: ρ*_Spearman_ = 0.24, *p* = 2.71 × 10^−20^) and VIS AT (*n*_pairs_ = 1 578; *UCP1: ρ*_Spearman_ = 0.49; *p* = 3.72 × 10^−96^, Figure 9H; *TMEM26: ρ*_Spearman_ = 0.45; *p* = 8.46 × 10^−81^; *UCP3: ρ*_Spearman_ = 0.62; *p* = 4.99 × 10^−165^; *NRG4: ρ*_Spearman_ = 0.35; *p* = 4.61 × 10^−46^). Together, these results strongly support the hypothesis that elevated levels of *MB* expression may be a characteristic of thermogenic adipocytes and linked to the browning of WAT in humans.

## 3 DISCUSSION

Prior studies reported gene expression of the hemoprotein myoglobin in murine BAT that can be further induced by cold exposure.^25^, ^26^, ^38^ Also in‐depth analysis of brown and white adipocyte transcriptomes revealed significantly higher (∼200‐fold) Mb gene expression in brown compared to white adipocytes.^38^ We here demonstrate that MB expression in BAT is highly induced during brown adipocyte differentiation and further increased during cold exposure in vivo.^28^ Previous studies reported initial phenotypic and metabolic alterations in MB‐deficient BAT of Mb‐KO mice and suggested a functional role of MB in BAT.^27^, ^28^ Our findings here demonstrate the dramatic effect of increasing MB expression for the metabolic activity of brown and white adipocytes in vitro and for proper BAT activity and thermoregulation in vivo.

Understanding the regulatory mechanisms controlling MB expression in BAT may enable the targeting and manipulation of MB expression pharmacologically. Acute treatment of brown adipocytes with PPARG agonists (rosiglitazone or free fatty acids) did not induce or change *Mb* gene expression. Adrenergic stimulation showed distinct regulation of MB expression in vitro (significant reduction on gene and protein level) and in vivo (increase in WAT with no changes in BAT after 1 week of CL‐treatment). Similar observations have been made for genes related to de novo lipogenesis in WAT,^39^ and we can similarly conclude that, while *Ucp1* expression is readily and cell‐autonomously induced by adrenergic signalling, other pathways or stimuli seem to be required in addition to inducing *Mb* expression in brown adipocytes in vitro. We did not observe a previously reported cell‐autonomous effect of hypothermia (exposure to 10°C) on *Mb* expression in isolated adipocytes,^27^ but have only cultured cells at 30°C. Also, stimulation of cold‐sensing receptor TRPM8 did not alter *Mb* expression. In muscle, a network of transcriptional factors regulate myoglobin gene expression^40^ with a key role for a PGC1a‐Calcineurin/MEF2 axis.^41^ Acting as a coactivator of nuclear receptors such as Nrf1, PGC1a is also increased in muscle and BAT upon cold‐exposure to stimulate mitochondrial biogenesis and adaptive thermogenesis^32^, ^42^ and may thus be involved in controlling MB expression during BAT activation. Knockdown of *Pgc1a* or *Nrf1* significantly reduced MB protein levels in imBA cells, indicating the key role of the PGC1a‐NRF1 axis in BAT MB expression. In line with these findings, our pilot investigation of *Mb* promotor methylation in response to cold exposure indicated epigenetic changes at a putative NRF1 binding site, which may facilitate expression in concert with increased PGC1a‐NRF1 activity.

The absence of MB in brown adipocytes results in limited mitochondrial respiratory capacity and decreased lipolytic response to adrenergic stimulation in vitro. As already indicated by respiratory rate measurements in BAT explants of Mb‐KO mice,^27^ we show a clear MB expression level‐dependent increase in maximal mitochondrial respiration and capacity in differentiated adipocytes, using immortalized and primary adipocytes expressing no (primary brown adipocytes of Mb‐KO), low (siRNA‐transfected imBA) or high levels (MB‐overexpressing imBA) of MB. Responsiveness to adrenergic stimulation of these adipocytes was similarly affected, with lower or missing MB expression dampening PKA activation and lipolysis, whereas overexpression of MB resulted in more pronounced PKA activation, lipolysis and FA release. Within brown adipocytes, we found the majority of MB in the cytosolic fraction, but also clear evidence for mitochondrially localized MB. In the mitochondria, we found evidence for MB in the OMM and in part also on the matrix side of the IMM. Whether the IMM localized MB contributes as an oxygen donor to complex IV (as shown in skeletal muscle^19^) remains to be further investigated. However, conditions of hyperoxia in cell culture and during mitochondrial respiration measurements indicate that major contributions of MB via increased O_2_ supply are likely not underlying the role of MB expression in adipocytes. Also, the content of MB protein we determined in differentiated brown adipocytes and BAT is 1‐2 orders of magnitude lower than in rat or human muscle (1‐20 μg/mg dry weight;^43^, ^44^). These amounts are likely not sufficient to substantially increase the O_2_ storage/buffering capacity of the cell.^33^, ^45^

We also observed no effects of MB expression on ROS and superoxide levels in imBA adipocytes. The most intriguing property of MB in the context of BAT is the reported lipid binding capacity.^20^ To investigate whether lipid binding is important for the observed beneficial effects of BAT MB, we first generated mutant MB based on information from molecular dynamic simulations.^34^ Simple dot blot lipid overlay assays confirmed reduced FA binding of mutant MB. Importantly, overexpression of this mutant MB in imBA adipocytes did not alter adipocyte metabolism and mitochondrial respiration. We, therefore, conclude that lipid binding seems to underlie MB contribution to enhanced substrate flux in brown adipocytes resulting in increased metabolic activity. The MB overexpressing brown adipocytes seem to adapt to this situation with increased mitochondrial and thermogenic gene and protein expression, supporting a feedback loop between MB and master regulator PGC1a to adjust mitochondrial biogenesis and respiratory capacity. To the best of our knowledge, this is the first time that MB lipid binding has been shown to be of functional relevance.

In vivo, MB deficiency translated into impaired thermoregulation when housing Mb‐KO mice at sub‐thermoneutral ambient temperatures and limited response to pharmacological BAT activation. Here, decreased expression of thermogenic and mitochondrial genes and reduced PKA signalling in brown adipocytes seem to be the underlying mechanisms resulting in lower BAT surface and body temperatures. At the gene expression level, differences in BAT after cold exposure between Mb‐KO and NMRI WT mice reflected the alterations we observed in brown adipocyte metabolism in vivo and after knockdown or knockout of *Mb* gene expression in vitro. Induced genes after cold exposure were related to the response to ROS in BAT of Mb‐KO mice, indicating the contributions of MB in scavenging ROS in active BAT in vivo. In vitro, we did not observe the effects of MB overexpression on ROS generation in imBA. Expression of *c‐Jun* was significantly increased in BAT of Mb‐KO, which represses basal and PKA‐induced *Ucp1* expression during brown adipocyte differentiation and after adrenergic stimulation,^46^ and may contribute to lower *Ucp1* expression in Mb‐KO BAT. Also, the significant reduction of *Trfc* expression reflects the less thermogenic character of Mb‐KO BAT compared to NMRI WT mice. *Tfrc*‐mediated iron uptake is an important regulator of thermogenic capacity in BAT, while independent from iron status *Trfc* also promotes brown adipocyte lineage commitment.^47^ Lower *Trfc* expression has also been observed in the muscle of Mb‐KO mice^48^ though functional relevance remains unclear. We did not find genes related to NO‐metabolism, and together with previously reported unaltered expression of NO synthases in BAT of Mb‐KO mice, this further indicates that MB may not significantly contribute to NO homeostasis in BAT.^27^ Together, loss of MB in BAT results in gene expression patterns reflecting altered substrate flux and ROS generation that together limit mitochondrial respiration and negatively affect BAT thermogenesis.

At the tissue level, cold‐induced PKA substrate and HSL phosphorylation were reduced, as was protein expression of UCP1, CYCS and COXIV. Mice showed clear adaption of BAT morphology to ambient temperatures with significantly decreasing lipid droplet size at lower temperatures, but no differences were observed between the genotypes. In line with previous reports, relative white AT mass was significantly higher in Mb‐KO, and also relative BAT mass was higher, which may reflect the whitening of BAT, but was not seen before.^27^ It has to be noted, that we investigated male mice in this study, while other studies found BAT morphology and phenotypic differences more pronounced in female mice.^27^, ^28^ Yet, we did not observe obvious differences in OCRs during Seahorse experiments using primary brown adipocytes from male or female NMRI WT and Mb‐KO mice. In line with previous data,^48^ we did not observe significant genotype‐specific differences in EE in animals held at thermoneutrality or mild cold stress, but indeed observed a significant genotype effect on EE after cold exposure and Mb‐KO mice also showed a blunted response to acute pharmacological adrenergic activation.

We clearly acknowledge two limitations of our in vivo studies. First, we analyzed whole‐body Mb‐KO mice, that were initially backcrossed for seven generations onto the NMRI background,^49^ using non‐littermate NMRI WT mice as controls. As mentioned above, we observe consistent findings regarding basic AT morphology, mass and BAT thermogenic gene expression comparing Mb‐KO to Mb‐WT mice, as reported by Aboouf et al.,^27^ using the same Mb‐KO on NMRI background, and Ono‐Moore et al.,^48^ using Mb‐KO mice on the C57BL/6N background. While the NMRI WT does not represent ideal controls, they still allow for a principle comparison. Also, the whole‐body Mb knockout itself does not represent an ideal model due to various systemic compensatory effects^29^ that may obscure or override BAT‐specific effects. Nevertheless, these proof of principle studies already indicate that expression of MB in BAT likely affects whole‐body energy expenditure in vivo, with future studies using conditional (B)AT‐specific Mb knockout mice necessary for confirmation.

We further demonstrate that MB expression in white adipocytes has similar metabolic effects as in brown adipocytes. While beige (or brite) and brown adipocytes have different developmental origins, RNAseq data showed that similar to brown adipocytes, beige adipocytes have significantly higher (∼12‐fold) *Mb* expression compared to white adipocytes in mice.^38^ We have here investigated for the first time *MB* expression in human VIS and SC white AT samples from well‐phenotyped individuals of the large Leipzig obesity biobank. We found that in human WAT, *MB* expression is higher in AT samples that show a higher thermogenic character (high expression of *UCP1* and other markers of browning). The inverse regulation of *MB* expression in SC and VIS AT with increasing BMI may reflect the different metabolic or lipolytic activity of the depots^50^ or also contribute to the different risk profiles of AT deposition in obesity.^51^ The higher lipolytic activity of VIS AT may warrant higher *MB* expression compared to SC AT (as observed in lean patients) with SC AT adapting to increasing fat accumulation and obesity with higher *MB* expression. On the other hand, reduced *MB* expression in VIS AT of obese patients may contribute to or reflect adipocyte dysfunction.

Previous studies had reported that the relationship between MB expression and BAT phenotype may be modulated to some degree by sex in mice.^28^ In this study, we did not observe gender‐specific differences, for example, using primary brown adipocytes from male or female Mb‐KO mice.

In our human data, the correlations of *MB* with waist circumference (in SC AT) and body fat (inverse in VIS AT) were more pronounced in female patients, yet remained significant after adjustment for sex. This indicates that there likely is no gender‐specific difference in AT *MB* expression, but rather a distribution effect with more lean control samples in the female subgroup of the cohort. In conclusion, we report for the first time that human *MB* is differentially expressed in SC and VIS AT depots, differentially regulated by the state of obesity, normalized after bariatric surgery and higher expressed in AT samples that exhibit a higher thermogenic potential. While these observations suggest a role of MB in human metabolic regulation, we acknowledge that from these correlations, conclusions regarding the causality or functional relevance of human adipose MB in metabolic disease mechanisms cannot be drawn. In the future, BAT‐ and AT‐specific Mb‐KO mouse models will help to clarify the contributions of MB deficiency in BAT or beige adipocytes of WAT to whole‐body energy expenditure in vivo.

Taken together, our studies establish myoglobin as a previously unrecognized and important regulatory element in BAT metabolism. We show that lipid binding seems to be the most important property of MB to enhance substrate flux and increase mitochondrial respiratory capacity, crucial for rapid adaptation to metabolic changes and (B)AT thermogenesis.

## 4 METHODS

### 4.1 Animal studies

Whole‐body Mb‐KO mice on the NMRI background were previously generated^29^, ^49^ and bred at the Sächsische Inkubator für Klinische Translation (SIKT), Leipzig. NMRI WT controls were obtained from Janvier (Saint Berthevin, France), and kept in the local animal house for one week for acclimatization. C57BL/6N mice were bred at the SIKT, Leipzig. All mice were housed in pathogen‐free facilities (3‐5 mice per group and cage) at 23°C on a 12 h light/dark cycle, as indicated. All mice were fed a standard chow diet (EV153, 3.3% from fat, Ssniff^®^, Soest, Germany) and had ad libitum access to water and food, except when indicated. Male NMRI WT and Mb‐KO mice (10‐12 weeks of age) were then adapted to single housing and subsequently housed in a climate chamber at either 30°C, 23°C or 8°C, respectively, for one week. Body temperature was measured after 5 days. After 7 days, BAT and tail surface temperatures were measured by thermal imaging (VarioCAM^®^ hr, Infratec, Dresden, Germany) as previously described.^52^ Acute cold‐tolerance test was done with single‐housed mice, which were transferred from 30°C to a climate chamber (Memmert HPP750 life) at 8°C for 6 h and rectal body temperature was measured every hour.

#### 4.1.1 Indirect calorimetry

Energy metabolism at different ambient temperatures was analyzed by indirect calorimetry using CaloSys V2.1 metabolic chambers (TSE Systems, Bad Homburg, Germany) at an age of 12 weeks as previously described.^53^ Food and water intake, mean O_2_ consumption, CO_2_ production, energy expenditure and spontaneous locomotor activity (X, Y, Z cage movement) were recorded every 5 min for 5 days at 23°C, followed by 5 days at thermoneutrality (30°C) and then 5 days at 8°C. Temperature ramps (from 23°C to 30°C and 30°C to 8°C) were done during the 12 h light phase. Data from four full days were analyzed for every temperature, after an initial 2 days of acclimatization or 24 h after each change of temperature. Energy expenditure data were analyzed using ANCOVA with body weight as a covariate as previously described.^54^ To analyze non‐shivering thermogenesis capacity, 12 weeks old male NMRI WT and Mb‐KO mice housed at 23°C were acclimated to chambers for one day. Then basal energy expenditure was measured for 60 min, before injecting mice subcutaneously with 1 mg/kg of β‐adrenergic agonist CL316,243 (CL) and measuring O_2_ consumption and CO_2_ production (1 min interval) for at least 60 min after injection. Chronic pharmacological activation of thermogenesis was induced by daily intraperitoneal injections of CL (1 mg/kg) for 10 days in male C57BL/6N mice. After sacrificing, organs were harvested, weighed (liver, subcutaneous inguinal (iWAT), epididymal (eWAT) and BAT) and processed for histological and biochemical analyses or snap frozen in liquid nitrogen.

#### 4.1.2 Histological analyses

AT histology, measurements of lipid droplet and adipocyte size distributions as well as immunohistochemical analyses were performed as previously described.^53^, ^55^ Immunohistochemistry was done using rabbit anti‐UCP1 polyclonal antibody (ab10983, Abcam, Cambridge, UK), anti‐MB antibody (ab77232) and HRP‐conjugated anti‐rabbit antibody (Dako Envision™+; Dako, Jena, Germany) and images were taken using a Keyence BZ‐X800 fluorescence microscope.

#### 4.1.3 DNA methylation analyses

Genomic DNA was extracted from 20–40 mg BAT tissue using GenElute^TM^ Mammalian Genomic DNA Miniprep Kit (Sigma‐Aldrich, USA). 300 ng of extracted DNA was further modified using Qiagen EpiTect Fast DNA Bisulfite Kit (Qiagen, Hilden, Germany) according to the manufacturer's protocols in order to determine CpG methylation by pyrosequencing. Analysis of CpG methylation was performed as previously described with modifications.^56^ Briefly, a CpG assay including four CpG sites located in a potential NRF1 transcription factor binding site within the promoter region of *Mb* was created using PyroMark Assay Design Software 2.0 (Qiagen). The genomic sequence of transcript NM_001164047 was derived from the UCSC genome browser (https://genome.ucsc.edu/) with mouse genome assembly GRCm39/mm39. Potential transcription factor binding sites were identified using the open‐access JASPAR database (https://jaspar.genereg.net,^57^). Primer sequences are listed in Table S1. The amount of 20 ng bisulphite‐treated DNA per sample was PCR amplified and the subsequent pyrosequencing analysis was run on a PyroMark Q24. The obtained results were analyzed via PyroMark Q24 software, version 2.0.6 (Qiagen). Each sample was PCR amplified and analyzed twice on different plates for replication purposes. Two no‐template controls per plate containing water as well as bisulphite treatment controls included in the CpG Assays were used for quality control. Only samples that reached “passed quality” during the pyrosequencing run were taken forward. The coefficients of variance (CVs) for replicates over all samples vary from 0.023 to 1.059. The mean methylation of replicates per CpG site as well as the mean methylation level across all four CpGs were calculated and used for statistical analyses.

### 4.2 Adipocyte cell culture and experiments

#### 4.2.1 Adipocyte differentiation

3T3‐L1, immortalized brown adipocytes (imBA, originally reported in^58^) as well as primary brown and white adipocytes (from male and female C57BL/6N, NMRI and Mb‐KO mice, as indicated) were cultured and differentiated into mature adipocytes as previously described.^53^

#### 4.2.2 Adipocyte transfection

MB overexpressing imBA and 3T3L1 were generated by stable transfection using a human MB (wildtype and mutants MB_K45A/K63A, MB_F46W/H64W and MB_ K45A/F46W/K63A/H64W) containing pcDNA3.1(+) plasmids (Genescript, Piscataway, NJ, USA) via nucleofection (transfection program CM‐137, 4D‐Nucleofector, Lonza, Basel, Switzerland). Empty vector‐transfected cells served as controls. Transfected cells were cultured under antibiotic selection. siRNA‐based knockdown of *Mb, Pgc1a* and *Nrf1* in imBA cells was performed as previously described.^59^ Briefly, imBA were cultured and differentiated in 15 cm petri dishes. On day 6 of differentiation, 1 × 10^6^ cells were seeded in 6‐well plates for gene expression and 500,000 cells into 12‐well plates for protein expression analysis. Cells were reverse transfected with 5 μl/ml Lipofectamine^®^ RNAiMAX reagent (#13778100, Thermo Fisher, Waltham, MA, USA) and Silencer Select siRNA (Mb #s69638, Pgc1a #s72017, Nrf1 #s70792 and control #4390843, Ambion, Kassel Germany) in final concentrations of 50 nM, respectively. Adipocyte differentiation was continued for three more days.

#### 4.2.3 Lipid droplet (LD) measurements

Adipocytes were seeded into 4‐well chamber slides (Sarstedt, Nümbrecht, Germany), differentiated and stained with AdipoRed Assay™ Reagent (Lonza) as previously described.^53^ Subsequently, cells were fixed in 4% PFA, washed, coated with Mowiol solution and sealed with a cover glass. Three images per well were taken using a Keyence BZ‐X800 fluorescence microscope. LDs in imBA clones were identified and separated with the cell count function of the BZ‐X800 Analyzer (upper/lower limit: 170 μm / 1 μm; areas at screen edges were removed). Incorrectly separated LDs were manually removed from the analysis. In primary brown adipocytes and 3T3‐L1 clones, LDs were automatically analyzed using Ilastik^60^ and CellProfiler.^61^ Briefly, a pixel‐based classifier was trained in Ilastik. Using the trained classifier, a probability map of each image was generated and loaded in CellProfiler. LDs were detected using the “IdentifyPrimaryObjects” module and areas were measured. For one well, the average LD area was calculated as the mean of three images.

#### 4.2.4 Proliferation assay

Cell proliferation and viability were determined using WST‐1 cell proliferation kit according to the manufacturer's protocol (#11644807001, Roche, Basel, Switzerland).

#### 4.2.5 Signal transduction assays

Differentiated adipocytes were treated with or without β‐adrenergic agonist CL316,243 (CL; 1 μM for 15 min), or adenylyl cyclase agonist forskolin (FSK; 1 μM for 15 min) respectively, prior to Western blot analysis.

#### 4.2.6 In vitro lipolysis, lactate release and ROS assay

Lipolysis was assessed in differentiated adipocytes under basal conditions and after the addition of CL or ISO (final concentrations of 100 nM) for 3 h. Glycerol release was measured using a lipolysis assay kit (ab185433, Abcam) according to the manufacturer's protocol. Free fatty acids (FAs) were measured in supernatants of differentiated adipocytes stimulated with CL or ISO (final concentrations 1 μM or 100 nM) for 3 h according to manufacturer's protocol (NEFA‐HR(2) Assay, Fujifilm Wako Chemicals Europe, Neuss, Germany). Supernatants of differentiated brown adipocytes, pre‐treated with or without 100 nM or 5 μM CL for 60 min, were collected and cellular l‐lactate release was measured using standard colourimetric assay as described in the manufacturer's protocol (ab65331, Abcam). Intracellular superoxide and ROS levels in differentiated brown adipocytes, pre‐treated with or without 1 μM CL for 60 min, were measured using a standard assay kit (ab139476, Abcam).

#### 4.2.7 Oxygen consumption assays

3T3‐L1, imBA or primary brown adipocytes were seeded into 24‐well V28 Seahorse assay plates at 22000 cells per well, differentiated as described above and assays were performed as published with minor modifications.^62^ Differentiated adipocytes were washed with assay medium (10 mM glucose, 2 mM L‐glutamine, 1 mM sodium pyruvate in Seahorse XF base medium at pH 7.4) and then incubated in assay medium for 45‐60 min at 37°C at low CO_2_. For mitochondrial stress tests, OCRs were measured in a Seahorse XFe24 analyser after the following injections: imBA and primary brown adipocytes: 2 μM oligomycin (Oligo), 2 μM FCCP, and 1 μM rotenone/antimycin A (Rot/AA) (103015‐100, Agilent); 3T3‐L1 cells: 2 μM Oligo, 1 μM FCCP, and 0.5 μM Rot/AA. For β‐adrenergic or adenylyl cyclase agonist treatment, 10 μM CL, 10 μM ISO or 10 μM FSK were acutely injected between Oligo and FCCP injections. Data were analyzed using the Wave software.

### 4.3 Mitochondrial content, mitochondrial isolation and mitochondrial protease protection assay

Assessment of mitochondrial content was done as previously described.^63^ In brief, DNA was extracted using the Allprep^®^ DNA/RNA Kit (Qiagen, Hilden, Germany) and a specific region of mtDNA (non‐NUMT) was amplified in a qPCR reaction. The nuclear gene *B2m* was used for normalization. Mitochondria from differentiated imBA_Ctrl adipocytes and imBA_hMB clones were isolated using a mitochondria isolation kit (#89874, Thermo Fisher). A mitochondrial protease protection assay using protease K to investigate mitochondrial integration of MB in differentiated imBA adipocytes was done as previously described^18^ with minor modifications. Mitochondria from differentiated imBA_Ctrl and imBA_hMB adipocytes were isolated in solution A, followed by two rounds of ultrasound sonification for 15 s each. In total, 200 μg crude mitochondria were used for the assays. Under the assay conditions, protease K (at 20 μg/ml) is not able to digest intra‐mitochondrial proteins without additional permeabilization of the OMM using either Triton‐X‐100 (TX100) or OS treatment, the latter preserving the mitoplasts/IMM. TOM20 (OMM proteins), Mitofilin (MIC60, IMM protein) and PDH (matrix protein) served as controls for the treatments. Differentially treated subfractions of isolated mitochondria were analyzed by Western blot.

### 4.4 RNA preparation, quantitative real‐time‐PCR (qPCR) and microarray analysis

RNA isolation from BAT was done using the RNeasy Lipid Tissue Mini kit (Qiagen, Hilden, Germany) as specified by the manufacturer. qPCR was performed using the LightCycler System LC480 and LightCycler‐DNA Master SYBR Green I Kit (Roche, Mannheim, Germany). Adipocyte gene expression was calculated by ΔΔCT method and normalized to *Nono* or *36b4* levels in each sample, as indicated.^64^ Primer sequences are listed in Table S2.

Microarray analysis (Clariom S Assay, mouse, Thermo Fisher) of BAT gene expression in cold‐challenged (6 h at 8°C, *n* = 5 per group) male Mb‐KO and NMRI WT mice was conducted at the Core Unit DNA Technologies (Core Facilities of the Faculty of Medicine; University of Leipzig) and data processing and analysis were done as previously described.^65^ Briefly, raw data were preprocessed according to the oligo Bioconductor R package (v1.50.0,^66^). The R packages oligo, Biobase (v2.46,^67^), and arrayQualityMetrics were used for data quality control (v3.42,^68^). Differentially expressed genes (DEGs) between NMRI WT and Mb‐KO in BAT were screened using the linear models for microarray data (LIMMA) method implemented in the limma (v3.42,^69^) statistical R package. To reduce the signal‐to‐noise ratio, array weights are included in the linear model. Only DEGs with an adjusted *p*‐value (_adj_ *p*) < .05 and a |log2 fold change (FC)| ≥ 0.5 were considered statistically significant (Table S3). A methodologically equivalent microarray analysis was performed in limiting the investigated gene set to genes from the following GO terms: GO:0000302 (response to ROS), GO:007173 (response to response to nitric oxide), GO:0090207 (regulation of triglyceride metabolic process), GO:0001666 (response to hypoxia), GO:0019216 (regulation of lipid metabolic process), GO:0005739 (mitochondrion), and GO:0005759 (mitochondrial matrix). Again, only DEGs with an _adj_ *p* < .05 and a |log2FC| ≥ 0.5 were considered statistically significant (Table S4). For smaller effects and a large gene set, some genes do not survive multiple testing, even though they have a strong effect on the conditions under study.

A broad gene list functional (KEGG 2019 mouse pathways) and GO enrichment analysis (biological process) were performed for all DEGs (_adj_ *p* < .05) irrespective of fold change using Enrichr (www.amppharm.mssm.edu/Enrichr ^70^; Tables S5–S10).

### 4.5 SDS‐PAGE, Western blot and ELISA

Proteins from cells or tissues were extracted with RIPA buffer (150 mM NaCl, 10 mM Tris (pH 7.2), 0.1% SDS, 1% TX100, 1% deoxycholate, 5 mM EDTA; supplemented with protease inhibitor cocktail (#11697498001, Roche) and phosphatase inhibitor cocktail (#4906845001, Roche)), subjected to SDS‐PAGE and transferred to nitrocellulose membranes using tank blot method as previously described.^56^ After incubation (overnight, 4°C) with primary antibodies, HRP‐coupled secondary antibodies were used and chemiluminescence was detected and quantified using the G:BOX Chemi XX9 system with GeneSys and GeneTools software (SynGene, Bengaluru, Karnataka, India). The following antibodies were used: from Cell Signaling Technologies, Danvers, MA, USA: phospho‐PKA substrates (RRXS*/T*) (#9624), COX IV (#4850), CYCS (#4280), SDHA (#9624), TUB1A (#2144), FASN (#3189S), ACLY (#432S), HSL (#4107S), pHSL (4126S), ADRB3 (ab94506), HSP90 (#4874), TOM20 (#42406S), anti‐rabbit‐HRP (#7074), anti‐mouse‐HRP (#7076); from Abcam: MB (ab77232), UCP1 (ab10983); from Sigma‐Aldrich, St. Louis, MO, USA: ACTB (#A1978), GAPDH (#G9545); from Thermo Fisher Scientific: OXPHOS (#45‐8099); from Proteintech, Rosemont, IL, USA: PDH (18068‐1); from BD Biosciences, Franklin Lakes, NJ, USA: CTNNB (61015).

Mouse and human MB in cell or tissue lysates were measured by Myoglobin SimpleStep ELISA (ab171580 and ab210965, Abcam) according to the manufacturer's instructions.

### 4.6 Dot blot fatty acid overlay assays

Binding of oleic and palmitic acid by wildtype MB and mutants MB_K45A/K63A, MB_F46W/H64W and MB_K45A/F46W/K63A/H64W (in the metMB and the oxygenated oxyMB form) were determined using a modified protein‐lipid overlay assay.^24^ Therefore, HEK293T cells were transfected with human MB plasmids as described above using electroporation. Cells were harvested 48 h after transfection, lysed in RIPA buffer and MB concentration was determined by ELISA as described above. Oxygenation of metMB was done by the addition of 3 mM sodium dithionite (Sigma, #157953) and subsequent oxygen exposure for 5 min.^71^ Palmitic and oleic acid (Sigma, #P0500 and #O1257) were solubilized in chloroform and 2 μl (containing 2 pmol to 400 nmol of fatty acid) were spotted on a 0.2 μm Amersham™ Protran^®^ Western‐blotting nitrocellulose membrane (#10600001, Cytiva, Marlborough, MA, USA) and air dried for 15 min. Solvent served as negative control and myoglobin‐containing lysates as respective positive controls. Membranes were blocked for 1 h using protein‐free blocking buffer (Thermo Fisher, #37572), followed by 15 min incubation with 1 μM metMB‐ or oxyMB‐containing lysates (for all variants). Then, membranes were washed twice for 10 min with TBS‐T (containing 0.05% Tween20) and incubated with anti‐MB antibody (Abcam ab77232, 1:1000 in TBS‐T) at 4°C overnight. Following two washing steps using TBS‐T, membranes were incubated with HRP‐conjugated anti‐rabbit antibody (Cell Signaling, #7074; 1:500 in TBS‐T) for 1 h at room temperature. After two final washing steps, enhanced chemiluminescence signals were detected using the G:BOX Chemi XX9 system.

### 4.7 Human data

#### 4.7.1 Simpson–Golabi–Behmel syndrome cells

Simpson–Golabi–Behmel syndrome (SGBS) cells were kindly supplied by Martin Wabitsch, University of Ulm, Germany and cultured in a basal medium consisting of DMEM/Ham F12 medium (Thermo Fisher) supplemented with 10% foetal bovine serum, 33 μmol/L biotin and 17 μmol/L pantothenic acid at 37°C with 5% CO2. Differentiation of SGBS cells into adipocytes was performed as previously described.^72^ Total RNA isolation, cDNA synthesis and quantitative real‐time PCR from SGBS cells and from AT samples of children were performed as previously described.^73^

#### 4.7.2 Leipzig childhood AT cohort

Children AT samples were collected as part of the Leipzig childhood AT cohort and were obtained from 67 Caucasian children (0‐18 years, 31 male, 36 female) undergoing elective surgery (orthopaedic surgery, herniotomy or orchiopexy, abdominal surgery etc.).^74^

#### 4.7.3 Leipzig obesity biobank (LOBB) cohorts

The adult cross‐sectional cohort comprises 2044 individuals from the LOBB. Omental VIS AT samples were collected from 1581 individuals with either normal weight (*n* = 58, mean age 60.5 ± 14.8 years, mean BMI 22.5 ± 1.9 kg/m^2^), overweight (*n* = 56, mean age 65.0 ± 12.7 years, mean BMI 27.2 ± 1.4 kg/m^2^) or obesity (*n* = 1467, mean age 47.1 ± 11.7 years, mean BMI 48.8 ± 8.4 kg/m^2^). Parallel paired abdominal SC AT samples were taken from 1013 individuals. In total, data from SC AT samples were included from 1475 individuals with either normal weight (*n* = 47, mean age 64.5 ± 13 years, mean BMI 22.9 ± 1.7 kg/m^2^), overweight (*n* = 56, mean age 62.7 ± 13.5 years, mean BMI 27.3 ± 1.5 kg/m^2^) or obesity (*n* = 1372, mean age 47.1 ± 12 years, mean BMI 48.8 ± 8.6 kg/m^2^).

The adult bariatric surgery cohort comprises paired samples of omental VIS and abdominal SC tissues from 43 individuals of the LOBB. AT biopsies were obtained from individuals with morbid obesity in the context of a two‐step bariatric surgery strategy, which in most cases included a sleeve gastrectomy as the first step and laparoscopic Roux‐en‐Y gastric bypass as the second step. Patients included in this study measured an average preoperative BMI of 53.4 ± 9.8 kg/m^2^ and an average postoperative BMI of 41.6 ± 7.7 kg/m^2^. All patients’ percentage of Excess BMI Lost (%EBMIL) was ≥40 % after surgery.

Adipose tissue samples were collected during elective laparoscopic abdominal surgery as described,^75^ were immediately frozen in liquid nitrogen and stored at −80°C until further analyses. Measurements of body composition and metabolic parameters were performed as previously described.^76^

RNA sequencing ‐ Single‐end and rRNA‐depleted RNA‐seq data for the adult cohorts were prepared with a SMARTseq protocol.^77^, ^78^ In brief, RNA was enriched and reverse transcribed by Oligo(dT) and TSO primers. ISPCR primers were used for cDNA amplification and cDNA was processed with Tn5 using a Nextera DNA Flex kit. All libraries were sequenced on a Novaseq 6000 instrument at the Functional Genomics Center Zurich (FGCZ). Fastp (v0.20.0^79^) was applied for the adapter and quality trimming of the raw reads (minimum read length of 18 nts, quality cut‐off of 20). The remaining reads were aligned using STAR (v2.7.4a^80^) against the human (GRCh38.p13) genome from GENCODE,^81^ allowing 50 multiple alignments per read. Standard quality control was conducted using FASTQC (v.0.11.4^82^). To assign genomic features to mapped reads, featureCounts (v2.0.1^83^) was applied, fractionating multiple mapped reads. Count data were homoscedastic normalized with respect to library size using the variance stabilizing transformation from DESeq2 (v1.32.0^84^). The adult cross‐sectional cohort was adjusted for reading depth and gender batch. Besides the read depth batch, the bariatric surgery cohort was adjusted for the patient ID batch as only paired samples were included in this cohort.

### 4.8 Statistical analysis

Data are presented as means ± SEM. Statistical analyses were performed using GraphPad Prism 9 (GraphPad, San Diego, CA, USA), except where indicated. Methods of statistical analyses were chosen on the basis of the design of each experiment and are indicated in the figure legends. Adjusted *p* < .05 was considered statistically significant.

The magnitude and significance of the correlation between body composition and metabolic parameter measurements and *MB* expression in the adult cohorts were calculated by a nonparametric statistical approach using the R package ggstatsplot (v0.9.1^85^) with the Spearman correlation coefficient and a confidence interval of 0.95. The ggstatsplot package was also used to examine comparisons of *MB* mRNA expression between subjects in the adult cohort or the child AT samples based on Kruskal–Wallis one‐way ANOVA and Dunn's test for pairwise comparisons. *p*‐values were corrected for multiple inferences using the Holm method.^86^ Analyses were performed under R version 4.1.

### 4.9 Data availability

Microarray data have been deposited in the ArrayExpress database at EMBL‐EBI (www.ebi.ac.uk/arrayexpress,^87^) under accession number E‐MTAB‐11059.

### 4.10 Study approval

#### 4.10.1 Animal studies

All animal experiments were approved by the local authorities of the state of Saxony (Germany), as recommended by the responsible local animal ethics review board (Landesdirektion Leipzig, Germany, TVV51/20; TVV26/16; T09/21).

#### 4.10.2 Leipzig childhood AT cohort

Written informed consent was obtained from all parents. The study was approved by the local ethics committee (Reg.No: 265‐08, 265‐08‐ff, University of Leipzig) and is registered in the National Clinical Trials database (NCT02208141).

#### 4.10.3 Leipzig obesity biobank (LOBB) cohorts

Written informed consent was obtained from all patients. The study was approved by the Ethics Committee of the University of Leipzig (approval no: 159‐12‐21052012) and performed in accordance with the declaration of Helsinki.

## CONFLICT OF INTEREST

The authors declare no conflict of interest.

## Supporting information



Supporting Information

Supporting Information
