# Supplementary material for: Myoglobin‐mediated lipid shuttling increases adrenergic activation of brown and white adipocyte metabolism and is as a marker of thermogenic adipocytes in humans
Source: Clin Transl Med. 2022 Dec 8;12(12):e1108. doi: 10.1002/ctm2.1108 (PMC9731393; doi:10.1002/ctm2.1108)
Supplement: Supplementary file 2 — Supporting Information [file CTM2-12-e1108-s001.docx]

**Myoglobin-mediated lipid shuttling increases adrenergic activation of brown and white adipocyte metabolism and is as a marker of thermogenic adipocytes in humans**

Lisa Christen, Helen Broghammer, Inka Rapöhn, Kevin Möhlis, Christian Strehlau, Aleix Ribas-Latre, Claudia Gebhardt, Lisa Roth, Kerstin Krause, Kathrin Landgraf, Antje Körner, Kerstin Rohde-Zimmermann, Anne Hoffmann, Nora Klöting, Adhideb Ghosh, Wenfei Sun, Hua Dong, Christian Wolfrum, Tienush Rassaf, Ulrike B. Hendgen-Cotta, Michael Stumvoll, Matthias Blüher, John T. Heiker^*^, Juliane Weiner^*^

**Supplementary Figure 1**

**Supplementary Figure S1.** Temperature-dependence of MB expression in BAT in male C57Bl/6N mice. A) *Mb* mRNA expression in epidydimal (eWAT), inguinal (iWAT) and brown adipose tissue (BAT), muscle (quadriceps femoris) and heart of male C57Bl/6N mice housed either at 30 °C or 8 °C for 1 week (n = 4 per group). For better appreciation, *Mb* expression in AT depots is also presented as insert. B) Direct comparison of Mb expression in BAT of 30 °C and 8 °C housed male and female C57Bl/6N mice. C) Representative images of MB immunohistochemistry in BAT from male C57Bl/6N mice as in (A). D) Time-resolved OCR of differentiated primary brown adipocytes from female NMRI WT and Mb-KO mice measured by Seahorse (representative experiment, n = 9/9). E) Quantification of basal respiration, proton leak, ATP production, acute response to FSK, maximum and spare respiratory capacity and non-mitochondrial respiration of samples in panel (D). Data are presented as mean ± SEM. Statistical significance was evaluated by multiple unpaired t-tests (A, B), corrected by the Holm-Šídák method (E). *p<0.05, **p<0.01, ***p<0.001. Scale bar: 400 µm.

**Supplementary Figure 2**

**Supplementary Figure S2.** Regulatory mechanisms of *Mb* expression in primary brown adipocytes and imBA. A) Effects of norepinephrine (NE) and CL316,243 (CL) on *Mb* mRNA expression in differentiated primary brown adipocytes. B-F) *Mb* gene expression in imBA cells cultured under normothermia or hypothermia (30°C) for 24 h (B), with menthol (C), with rosiglitazone (D) or various conentrations of linoleic acid (E) or oleic acid (F). Data are presented as mean ± SEM of at least two independent experiments.

**Supplementary Figure 3**

**
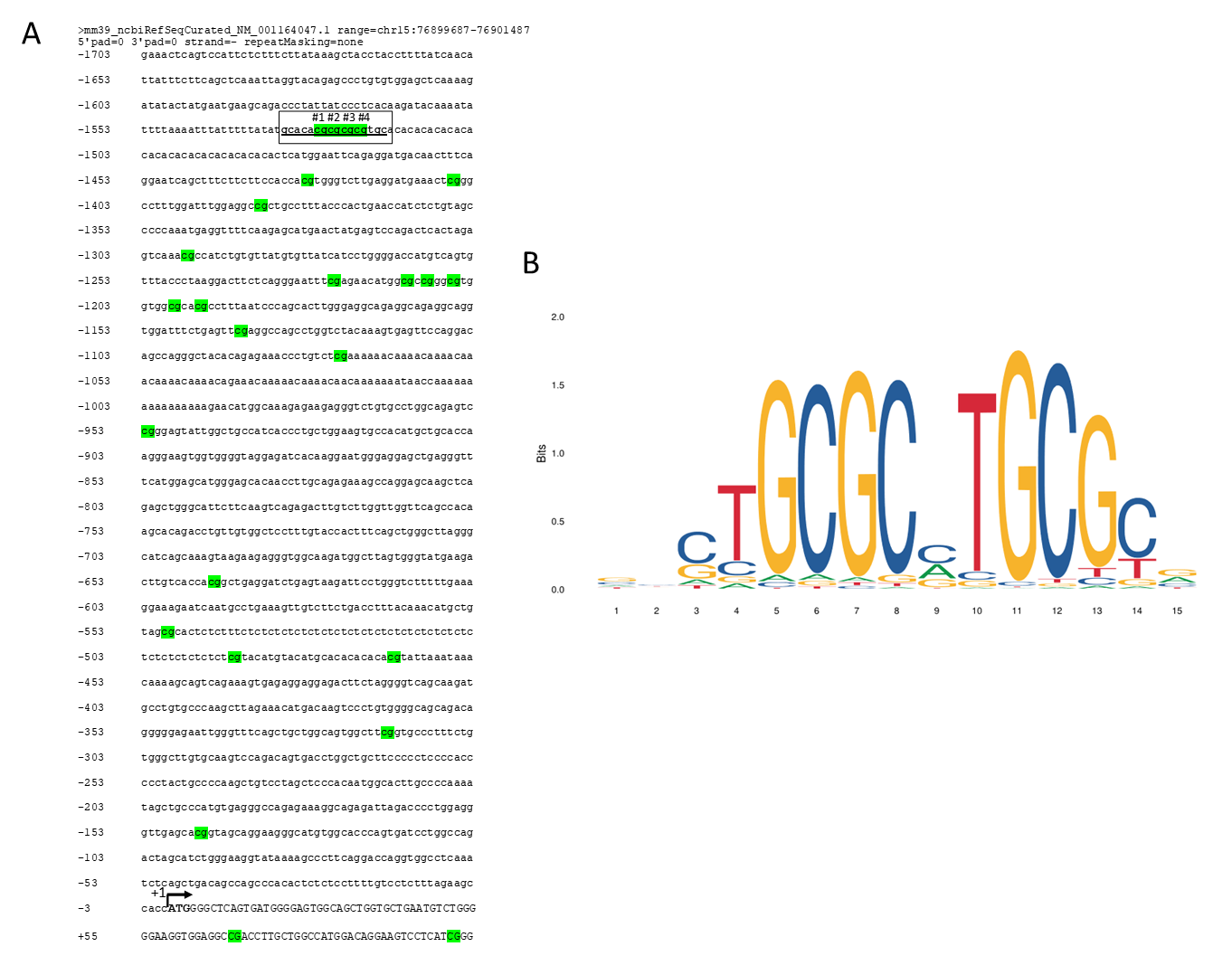
**

**Supplementary Figure S3.** Genomic Sequence of murine myoglobin transcript NM_001164047.1 promoter region. A) Sequence derived from UCSC genome browser (access January 2022), genome assembly mm39. Line numbering relative to translation start site (ATG) marked by a bent arrow and +1. CpG Assay Design Softare 2.0 (Qiagen) was used to create a PyroMark CpG Assay (underlined) including 4 CpG sites marked with # and numbered in consecutive order. The assay was designed for the use of PyroMark Q24 system (Qiagen). A potential Nrf1 transcription factor binding site is highlighted with a rectangle and was identified using the open access JASPAR database. B) Sequence logo of matrix profile MA0506.2 (Nrf1). To search for potential binding sites of Nrf1 within NM_001164047, the genomic sequence shown in (A) was loaded into open access JASPAR database (https://jaspar.genereg.net/, latest access January 2022). Four predicted binding sites for Nrf1 were identified with similarity score values of 9.5 to 10.5 with a threshold of 80% . Nrf1 = nuclear respiratory factor 1.

**Supplementary Figure S4**

**Supplementary Figure S4.** A) Cell viability was compared in imBA_Ctrl and imBA_hMB cultured for 24 h and formation of formazan dye was quantified. B) Expression of adipogenesis genes in adipocytes at day 0, 4 and 8 of differentiation. Gene expression is relative to imBA_Ctrl at day 0 and normalized to *Nono*. C) Time-resolved OCR of differentiated imBA_Ctrl and imBA_hMB adipocytes measured by Seahorse including FSK injection (representative experiment, n = 9/10). D) Mitochondrial content measured by mtDNA assay in differentiated imBA_Ctrl, imBA_hMB and imBA_hMB 4xmut adipocytes (n = 6/5/3). E, F) Western blot analysis and quantification of mitochondrial proteins SDHA, CYCS and COXIV in differentiated imBA_Ctrl and imBA_hMB adipocytes (day 8, representative of two experiments). G) Western blot analysis of cytosolic and membrane localized ADRB3 protein expression in differentiated imBA_Ctrl and imBA_hMB adipocytes. Upper panel: anti β-catenin antibody; middle panels: anti HSP90 antibody, anti ADRB3 antibody; lower panel: ponceau. H) Representative picture indicating increased acidification of cell culture media in imBA_hMB compared to imBA_Ctrl adipocytes. I) Western blot analysis of cytosolic and mitochondrial MB protein in pools of differentiated imBA_Ctrl and imBA_hMB adipocytes. Upper panel: anti SDHA antibody; middle panel: anti GAPDH antibody; lower panel: anti MB antibody. J) Quantification of lipid accumulation in imBA_Ctrl, imBA_hMB and imBA_hMB 4xmut adipocytes after differentiation. Data are presented as mean ± SEM of at least two experiments. Statistical significance was evaluated by unpaired t-tests (C). *p<0.05.

**Supplementary Figure S5**


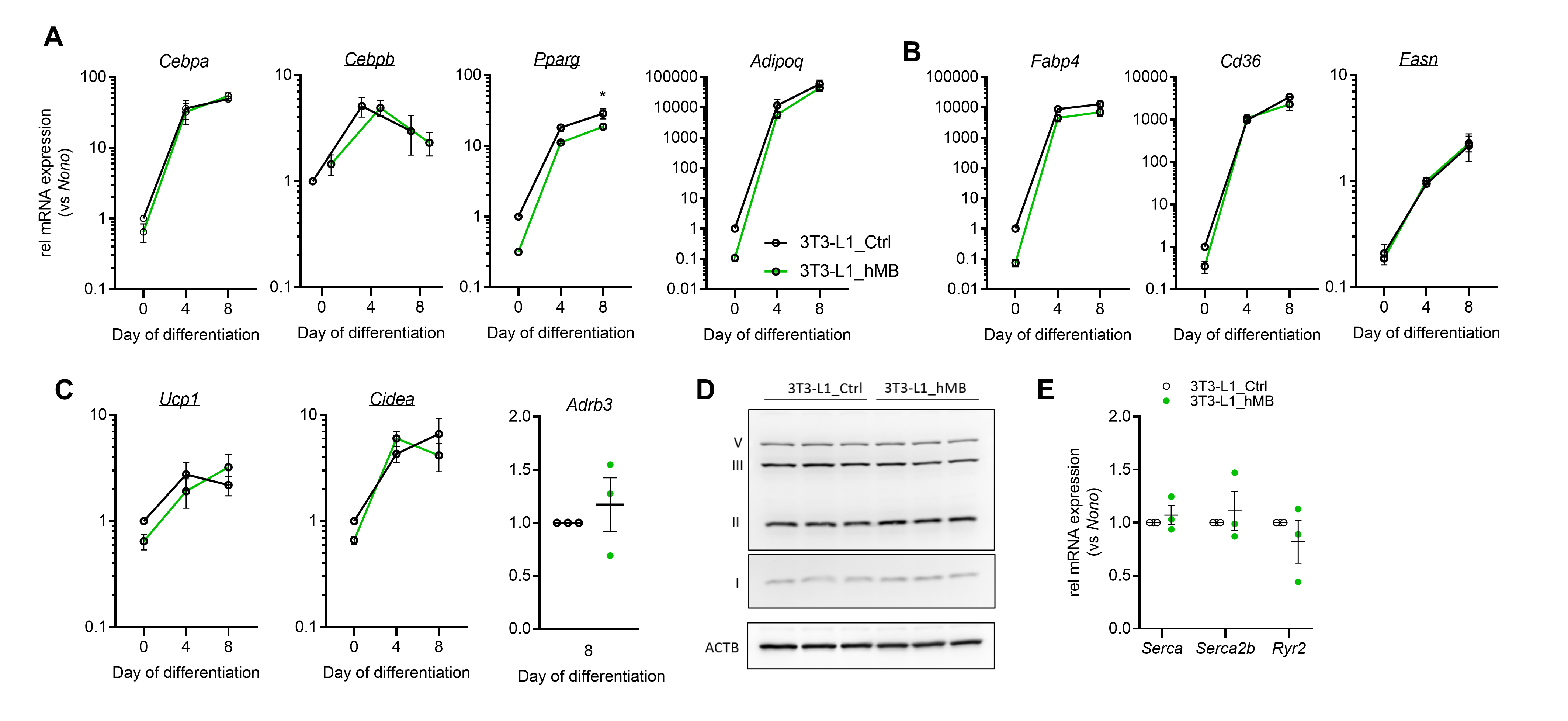


**Supplementary Figure S5.** A-C) Expression of marker genes of adipogenesis (*Cebpa, Cebpb, Pparg* and *Adipoq*, A), of lipid uptake (*Fabp4, Cd36* and *Fasn*, B) and thermogenesis (*Ucp1,* *Cidea* and *Adrb3*, C) in 3T3-L1_hMB and 3T3-L1_Ctrl adipocytes at day 0, 4 or 8 of differentiation. Gene expression is relative to 3T3-L1_Ctrl at day 0 and normalized to *Nono*. D) Westen blot analysis of OXPHOS protein expression (I: subunit NDUFB8; II: SDHB, III: UQCRC2, V: ATP5A) in differentiated 3T3-L1_hMB and 3T3-L1_Ctrl adipocytes. E) Expression of Ca-cycling genes in beige adipose thermogenesis (*Serca, Serca2b* and *Ryr2*) in differentiated 3T3-L1_hMB and 3T3-L1_Ctrl adipocytes. Gene expression is relative to 3T3-L1_Ctrl at day 8 and normalized to *Nono*. Data are presented as mean ± SEM of at least three experiments. *p<0.05.

**Supplementary Figure S6**

**Supplementary Figure S6.** A) Energy expenditure during light and dark phase of NMRI WT and Mb-KO mice housed at 8 °C or 30 °C during indirect calorimetry (averaged data of n = 6/5). B, C) Regression plots of EE against body weight with the ANCOVA test using body weight as covariate for mice housed at 23 °C or 30 °C (n = 6/5). D, E) Food intake (adjusted to body weight) and respiratory exchange rates (RER) of NMRI WT and Mb-KO mice housed at 30 °C, 23 °C or 8 °C for 1 week (n = 6/5). F, G) Locomotor activity (X+Y) and rearing (Z) of NMRI WT and Mb-KO mice during indirect calorimetry (n = 6/5). H, I) Relative organ weights of liver, and epididymal (eWAT), inguinal (iWAT) and BAT of NMRI WT and Mb-KO mice housed at 30 °C (H) or 23 °C (I) for 1 week (n = 6/4). J) Representative H&E-stained sections of BAT (40x), iWAT and eWAT (20x) from control and Mb-KO mice housed at 30 °C and 23 °C. K, L) Adipocyte lipid droplet size distribution in iWAT (K) and eWAT (L) from NMRI WT and Mb-KO mice shown in G (n = 5/4). Data are shown as mean ± SEM. Statistical significance was evaluated by two-way ANOVA with Šídák's post-hoc test or multiple uncorrected t-tests (H, I). *p-value < 0.05, **p-value < 0.01, ***p-value < 0.001.

**Supplementary Figure S7**

**Supplementary Figure S7.** A) Gene expression of thermogenic and mitochondrial genes in BAT of NMRI WT and Mb-KO mice housed at 8 °C for 1 week (n = 6/4). Gene expression is relative to NMRI WT and normalized to *36b4*. B) Quantification of OXPHOS complex protein expression from Western blot analyses in BAT of NMRI WT and Mb-KO mice housed at 8 °C for 1 week (n = 6/4). C) Western blot analysis (C) and quantification of PKA (D) and HSL (E) activation in BAT of NMRI WT and Mb-KO mice housed at 8 °C for 1 week (n = 5/5). Panels from top to bottom: anti-phospho-PKA substrate antibody; Tubulin antibody, pHSL antibody, HSL antibody, Tubulin antibody. F) Oxygen consumption before and up until 12 h after a single i.p. injection of CL in male NMRI WT and Mb-KO mice (n = 4/6). Data before and until 30 min after CL injection are shown in Figure 8G-H. G) Area under the curve of VO_2_ post CL injection shown in (F) and results of ANCOVA analysis. H) Expression of top DEG from microarray analysis in BAT of acutely cold-exposed NMRI WT and Mb-KO mice measured by qPCR (upregulated: *Ier3,* down-regulated: *Tfrc*, *Igf1* and *Adcy10* in Mb-KO BAT). Data are shown as mean ± SEM. Statistical significance was evaluated with multiple uncorrected t-tests. *p-value < 0.05, **p-value < 0.01.

**Supplementary Figure S8**


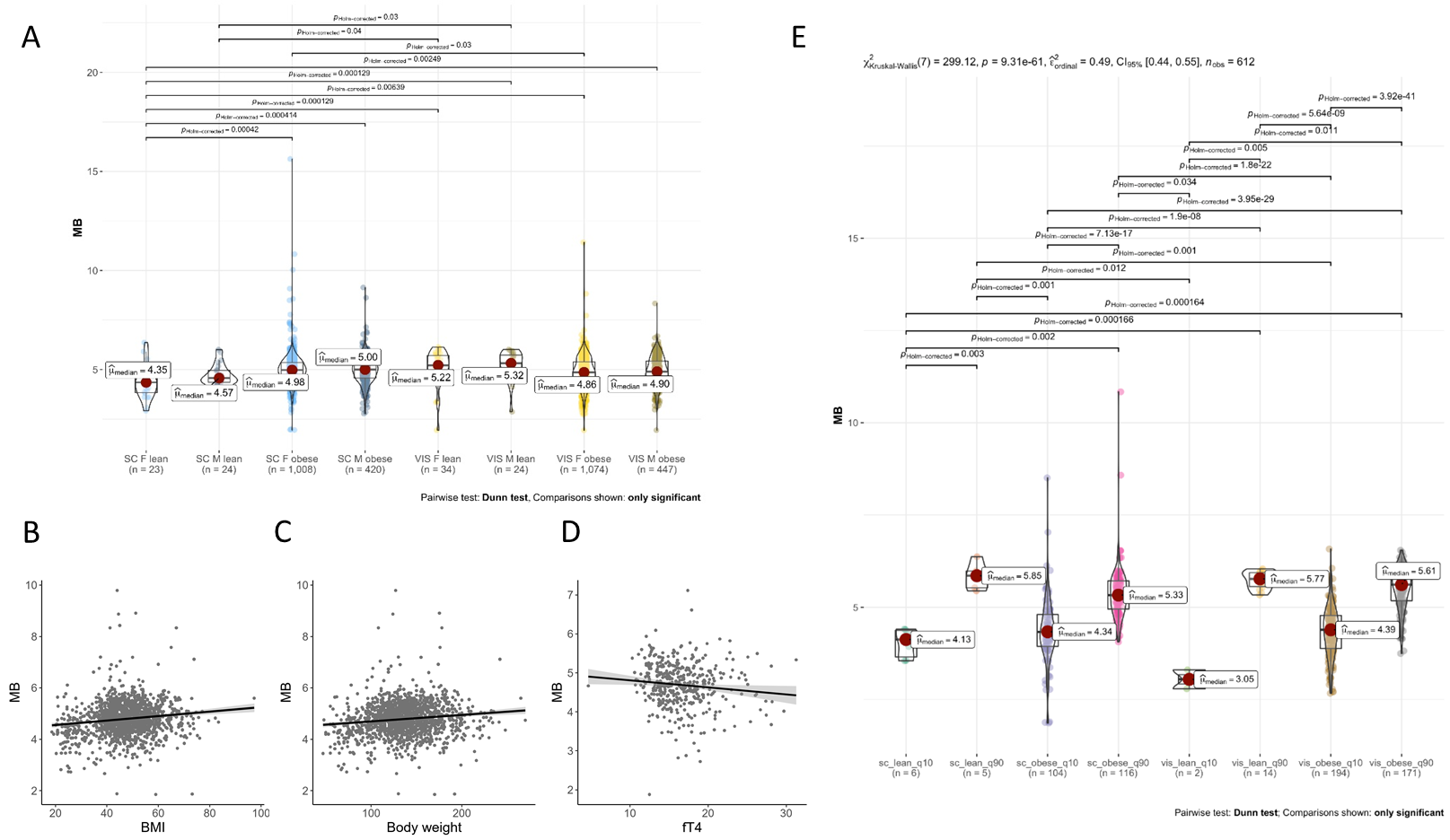


**Supplementary Figure S8.** A) Subcutaneous (SC) and visceral adipose tissue (VIS) *MB* gene expression in female and male BMI subgroups (normal weight (20 ≤ BMI ≤ 25), overweight (25 < BMI < 30) and obese (BMI ≥ 30)). B-D) Correlation of SC AT *MB* expression with BMI (B) as well as body weight (C) and inverse correlation with free T4 (D) in humans. E) *MB* gene expression in BMI subgroups of SC and VIS AT from patient samples belonging either to the 10^th^ quantile or 90^th^ quantile of AT *UCP1* expression. Statistical significance was evaluated by Kruskal-Wallis one-way ANOVA and Dunn's test for pairwise comparisons and corrected for multiple inference using the Holm method (A, E). p-values are given in the figure.
